# Comparative efficacy and safety of IL-23 p19 monoclonal antibodies for plaque psoriasis: a bayesian network meta-analysis of randomized controlled trials

**DOI:** 10.3389/fimmu.2026.1873488

**Published:** 2026-08-12

**Authors:** Tong Xin, Shanshan Sun, Yihui Liu

**Affiliations:** Shandong College of Traditional Chinese Medicine, Yantai, Shandong, China

**Keywords:** Bayesian network meta-analysis, efficacy, IL-23 p19 monoclonal antibody, plaque psoriasis, safety assessment

## Abstract

**Objective:**

To compare the efficacy and safety of IL-23 p19 monoclonal antibodies for moderate-to-severe plaque psoriasis.

**Methods:**

PubMed, Embase, Web of Science, and the Cochrane Library were searched from inception to 21 February 2026. We included randomized controlled trials of guselkumab, risankizumab, tildrakizumab, or mirikizumab. Efficacy outcomes were PASI75, PASI90, PASI100, and IGA/PGA/sPGA 0/1; safety outcomes were overall adverse events (AEs) and serious adverse events (SAEs). Risk of bias was assessed using RoB 2. A Bayesian network meta-analysis generated odds ratios (ORs) and 95% credible intervals (CrIs), with SUCRA, transitivity, heterogeneity, leave-one-out, and publication-bias assessments.

**Results:**

Thirteen articles comprising 15 RCTs or independent study units were included. Networks were largely placebo-centred, with limited direct active-agent comparisons. Versus placebo, all four agents significantly improved PASI75, PASI100, and IGA/PGA/sPGA 0/1. For PASI90, risankizumab, guselkumab, and mirikizumab were superior to placebo, whereas the tildrakizumab CrI crossed the null. PASI75 ORs versus placebo were 124.04 for risankizumab, 105.41 for guselkumab, 72.36 for mirikizumab, and 24.05 for tildrakizumab. Corresponding ORs for IGA/PGA/sPGA 0/1 were 89.73, 68.84, 52.82, and 21.46. Most comparisons among risankizumab, guselkumab, and mirikizumab were not statistically significant; tildrakizumab showed lower PASI75 and IGA/PGA/sPGA 0/1 responses. SUCRA generally ranked risankizumab highest or near highest, followed by guselkumab and mirikizumab, with tildrakizumab lower. No agent differed significantly from placebo in AE or SAE risk. Sensitivity analyses supported stable efficacy findings, and Egger’s tests found no clear publication bias.

**Conclusion:**

IL-23 p19 antibodies improve skin-clearance outcomes without a clear increase in overall AE or SAE risk. Risankizumab had the most favourable ranking probability, but limited direct evidence and overlapping CrIs preclude definitive superiority claims. Biologic selection should also consider prior treatment, comorbidities, dosing, access, and patient preference. Head-to-head trials and long-term real-world studies are needed.

**Systematic review registration:**

https://www.crd.york.ac.uk/PROSPERO, identifier CRD420261382915.

## 1 Introduction

Psoriasis is a chronic, recurrent, immune-mediated inflammatory skin disease, among which plaque psoriasis is the most common clinical subtype. It is primarily characterized by well-demarcated erythematous plaques, scaling, and varying degrees of pruritus or pain (1). Psoriasis is not merely a skin-limited disease; rather, it is closely associated with multiple comorbidities, including psoriatic arthritis, cardiovascular disease, metabolic syndrome, obesity, diabetes, and depression, thereby substantially increasing the long-term disease burden of affected patients (2). Global burden of disease studies have shown that the prevalence of psoriasis varies across regions and populations, yet its overall disease burden remains considerable and continues to receive increasing attention due to population growth, aging, and the growing need for chronic inflammatory disease management (3). For patients with moderate-to-severe plaque psoriasis, conventional systemic therapies and phototherapy remain clinically valuable but are often limited by insufficient efficacy, cumulative toxicity, contraindications, poor long-term adherence, or the need for intensive safety monitoring (4). Therefore, targeted biologic agents capable of achieving rapid, profound, and durable skin clearance while maintaining long-term safety have become an important component of treatment strategies for moderate-to-severe plaque psoriasis.

The IL-23/IL-17 immune axis plays a central role in the pathogenesis and progression of psoriasis. IL-23 maintains pathogenic Th17-cell responses and promotes the production of inflammatory cytokines such as IL-17 and IL-22, thereby driving abnormal keratinocyte proliferation and epidermal inflammation (5). Studies have shown that IL-23 levels are elevated in psoriatic lesional skin compared with non-lesional skin, suggesting that sustained activation of IL-23 signaling is one of the key mechanisms underlying chronic inflammation in psoriasis (5). Unlike blockade of the shared p40 subunit of IL-12/23, selective targeting of the IL-23 p19 subunit enables more precise inhibition of the IL-23 pathway, providing a mechanistic rationale for potent efficacy while preserving IL-12-related immune function as much as possible (6). Currently, IL-23 p19 monoclonal antibodies, including guselkumab, risankizumab, tildrakizumab, and mirikizumab, have been systematically evaluated in randomized controlled trials for moderate-to-severe plaque psoriasis. Previous reviews of IL-23 p19 inhibitors have shown that this drug class demonstrates strong efficacy and favorable safety in both clinical trials and real-world studies (7). Key phase III trials, including the VOYAGE, UltIMMa, reSURFACE, and OASIS programs, have demonstrated that IL-23 p19 monoclonal antibodies provide clear efficacy advantages over placebo in outcomes such as PASI75, PASI90, PASI100, and physician global assessment of clear or almost clear skin (8, 9). Previous safety studies and long-term follow-up data have also suggested that IL-23 p19 inhibitors are generally well tolerated, with low rates of serious adverse events and a favorable long-term therapeutic profile (10).

Although IL-23 p19 monoclonal antibodies have substantially changed the therapeutic landscape for moderate-to-severe plaque psoriasis, several unresolved issues remain regarding the comparative efficacy and safety of different agents (11). First, most pivotal clinical trials have compared a single IL-23 p19 monoclonal antibody with placebo or selected active comparators, whereas direct head-to-head evidence among guselkumab, risankizumab, tildrakizumab, and mirikizumab remains limited. Second, previous network meta-analyses have often evaluated TNF-α inhibitors, IL-17 inhibitors, IL-12/23 inhibitors, and IL-23 inhibitors as broad biologic categories, which may obscure efficacy differences within the IL-23 p19 monoclonal antibody subclass (4). As treatment goals for psoriasis have shifted from PASI75 toward more stringent skin clearance targets, including PASI90, PASI100, and IGA/PGA/sPGA 0/1, reliance on traditional efficacy endpoints alone is no longer sufficient to fully inform contemporary clinical decision-making. At the same time, because biologic therapy usually requires long-term maintenance, evaluating safety outcomes such as AE and SAE in addition to overall efficacy is essential for balancing clinical benefits against potential risks (10). Therefore, a network meta-analysis focusing specifically on comparisons within the IL-23 p19 monoclonal antibody class and incorporating multiple levels of efficacy endpoints together with safety outcomes is urgently needed to provide more targeted evidence.

Based on this rationale, the present study aimed to comprehensively evaluate the clinical benefits and safety of IL-23 p19 monoclonal antibodies for plaque psoriasis using a Bayesian network meta-analysis (BNMA). This study included randomized controlled trial evidence for guselkumab, risankizumab, tildrakizumab, and mirikizumab, and established a systematic comparative framework covering PASI75, PASI90, PASI100, IGA/PGA/sPGA 0/1, AE, and SAE. Compared with conventional pairwise meta-analysis, network meta-analysis can integrate direct and indirect evidence in the absence of sufficient direct head-to-head trials, thereby estimating the relative efficacy and safety of different IL-23 p19 monoclonal antibodies. In addition, this study incorporated transitivity assessment, heterogeneity analysis, SUCRA ranking, sensitivity analysis, and publication bias assessment to enhance the interpretability and robustness of the comparative findings. The results of this study are expected to clarify the relative advantages of different IL-23 p19 monoclonal antibodies in terms of deep skin clearance and safety, and to provide more precise evidence for individualized biologic selection in patients with plaque psoriasis.

## 2 Methods

### 2.1 Study design

This study was conducted in accordance with methodological standards for network meta-analysis (12), aiming to evaluate the clinical benefits and safety of IL-23 p19 monoclonal antibodies for moderate-to-severe plaque psoriasis and to compare the relative efficacy of different agents. The study population was restricted to adults with moderate-to-severe plaque psoriasis. The interventions were IL-23 p19 monoclonal antibodies, and the base-case comparator was placebo. The main efficacy outcomes included PASI75, PASI90, PASI100, and IGA/PGA/sPGA 0/1, while safety outcomes included overall adverse events and serious adverse events.

### 2.2 Literature search strategy

A systematic literature search was conducted in PubMed, Embase, Web of Science, and the Cochrane Library from database inception to February 21, 2026. The search strategy combined controlled vocabulary and free-text terms (13), and was developed around plaque psoriasis, IL-23 p19 monoclonal antibodies, and randomized controlled trials. The PubMed strategy used randomized-trial publication types and trial-related free-text terms rather than the full Cochrane highly sensitive RCT filter. The expression NOT (animals[mh] NOT humans[mh]) was used only to remove animal-only records and was not intended to exclude human studies. The reference lists of included studies were also manually screened to identify potentially eligible studies. The complete search strategies are provided in the **Supplementary File**. This study strictly followed current guidelines and relevant reporting standards for systematic reviews and meta-analyses, and was registered in PROSPERO under the registration number CRD420261382915.

### 2.3 Inclusion criteria

Studies were eligible for inclusion if they met all of the following criteria: patients were clinically diagnosed with plaque psoriasis, mainly adult patients with moderate-to-severe plaque psoriasis; the study design was a randomized controlled trial; the intervention was an IL-23 p19 monoclonal antibody, including guselkumab, risankizumab, tildrakizumab, or mirikizumab; the comparator was placebo in the base-case analysis; at least one of the following outcomes was reported: PASI75, PASI90, PASI100, IGA/PGA/sPGA 0/1, AE, or SAE; and the sample size and number of events in each study group could be extracted, or the corresponding effect size could be calculated from the original data. The inclusion criteria were defined according to the PICOS framework to ensure clarity in the research question, population, intervention, comparator, and outcomes.

### 2.4 Exclusion criteria

The following studies were excluded: non-randomized controlled studies, including observational studies, case reports, reviews, meta-analyses, systematic reviews, conference abstracts, or animal experiments; studies in which the population was not patients with plaque psoriasis, or in which the psoriasis subtype was inconsistent with the scope of this study; studies in which the intervention was not an IL-23 p19 monoclonal antibody, or in which the number of RCTs for the target drug was insufficient to form a network comparison; studies without a placebo group; studies with unavailable key outcome data that could not be obtained from the original article or supplementary materials; and duplicate publications or studies using overlapping data sources, for which the most complete or most recently published report was retained.

### 2.5 Study selection and data extraction

Study selection and data extraction were independently performed by two reviewers (TX and SS). Retrieved records were imported into EndNote reference-management software for duplicate removal, followed by title/abstract screening and full-text assessment to determine final eligibility. Disagreements between the two reviewers were resolved through discussion or adjudication by a third reviewer (YL).

Extracted data included first author, publication year, study name, study region, study design, sample size, intervention, comparator, dosing regimen, follow-up duration, mean age, sex distribution, additional baseline covariates where available (including baseline PASI score, body surface area involvement, BMI, disease duration, prior biologic exposure, prior systemic therapy, and previous treatment failure), main efficacy outcomes, safety outcomes, and adverse event reporting. Because these additional baseline covariates were incompletely reported across trials, they were considered qualitatively and were not presented as a complete arm-level dataset. For efficacy outcomes, the placebo-controlled induction assessment was preferentially used, primarily week 16, and trial-specific selected time points are summarized in **Supplementary Table 2**. For multi-arm or multi-dose studies, the prespecified target dose arm used in the network meta-analysis was extracted. Active comparator arms, such as adalimumab, ustekinumab, etanercept, or secukinumab arms, were not included in the base-case IL-23 p19 network because they represented different drug classes or non-placebo active comparators and would have changed the clinical scope and transitivity structure of the analysis. If a single article contained two independent RCTs, each trial was extracted and analyzed as an independent study unit.

### 2.6 Outcome definitions

The main efficacy outcomes were PASI75, PASI90, and PASI100, which indicate at least 75%, 90%, and 100% improvement from baseline in the Psoriasis Area and Severity Index (PASI), respectively. IGA/PGA/sPGA 0/1 was defined as a score of 0 or 1 on the Investigator’s Global Assessment, Physician’s Global Assessment, or static Physician’s Global Assessment, representing clear or almost clear skin. Safety outcomes included overall adverse events and serious adverse events. AE was defined as any unfavorable medical event occurring during the study period. SAE was defined as an event resulting in death, life-threatening conditions, hospitalization or prolonged hospitalization, significant disability, or other clinically important serious outcomes.

### 2.7 Risk of bias assessment

The methodological quality of included RCTs was assessed using the Cochrane RoB 2.0 tool (14). Five domains were evaluated: bias arising from the randomization process, bias due to deviations from intended interventions, bias due to missing outcome data, bias in measurement of the outcome, and bias in selection of the reported result. Each domain was judged as low risk of bias, some concerns, or high risk of bias, and an overall risk-of-bias judgment was then assigned. Risk of bias was independently assessed by two reviewers (TX and SS), and disagreements were resolved through discussion or adjudication by a third reviewer (YL).

### 2.8 Statistical analysis

A Bayesian network meta-analysis was performed to compare the efficacy and safety of different IL-23 p19 monoclonal antibodies. The modeling framework followed the generalized linear modeling approach proposed by Dias et al. (15). For each dichotomous outcome, a binomial likelihood with a logit link was used, and treatment effects were expressed as ORs with 95% CrIs. The R implementation used gemtc/rjags with the default weakly informative priors implemented in gemtc for treatment effects and between-study heterogeneity; no additional custom prior distributions were specified. For efficacy outcomes, an OR greater than 1 indicated a higher clinical response rate for IL-23 p19 monoclonal antibodies compared with placebo. For safety outcomes, an OR greater than 1 indicated a higher risk of adverse events.

Evidence networks were constructed using placebo as the common comparator. In the network plots, nodes represented interventions, node size reflected the sample size for each intervention, lines indicated direct comparative evidence, and line thickness reflected the number of direct comparisons. Network meta-analysis models were constructed separately for PASI90, PASI75, PASI100, IGA/PGA/sPGA 0/1, AE, and SAE. Full league tables for these outcomes are provided in **Supplementary Table 1**.

Bayesian models were estimated using Markov chain Monte Carlo methods with four chains, 20, 000 adaptation iterations, 50, 000 sampling iterations, and a thinning interval of 1. A random-effects consistency model was used as the primary model because the included trials differed in region, dosing schedule, induction time point, baseline characteristics, and study design. Convergence was evaluated using trace plots, posterior density plots, and the potential scale reduction factor. Model adequacy was assessed by residual deviance and the deviance information criterion where available. Statistical significance was determined using 95% CrIs, and a difference was considered statistically significant when the 95% CrI did not cross 1.

### 2.9 Assessment of transitivity

To evaluate the transitivity assumption of the network meta-analysis, key baseline characteristics and potential effect modifiers were compared across intervention groups. Transitivity is a core assumption of network meta-analysis and requires that effect modifiers be comparably distributed across different direct comparisons, as recommended by the PRISMA-NMA extension and related methodological literature (16). Mean age and male proportion were available with sufficient consistency for quantitative comparison. Additional baseline covariates were considered where reported, but incomplete reporting precluded a full quantitative comparison across all treatment nodes. Therefore, the transitivity assessment was interpreted cautiously rather than as definitive proof that all effect modifiers were balanced.

### 2.10 Heterogeneity analysis

Heterogeneity analysis was performed for four major efficacy outcomes: PASI90, PASI75, PASI100, and IGA/PGA/sPGA 0/1. Between-study variability and the potential influence of individual studies on the overall estimates were assessed by comparing study-specific effect estimates and their credible intervals. If individual studies showed markedly wide intervals or deviated from the overall distribution, sensitivity analyses were further considered to evaluate the robustness of the results.

### 2.11 Treatment ranking analysis

SUCRA and rankograms were used to assess the relative ranking of different IL-23 p19 monoclonal antibodies for the main efficacy outcomes (17). A higher SUCRA value indicated a greater probability that the intervention achieved a more favorable ranking for the corresponding outcome. Because ranking probabilities may be unstable when credible intervals are wide or active-agent comparisons are indirect, SUCRA values were interpreted together with pairwise effect estimates, CrIs, network geometry, and certainty of evidence rather than as standalone evidence of superiority.

### 2.12 Sensitivity analysis

A leave-one-out sensitivity analysis was conducted. Each individual study was sequentially excluded, and the network meta-analysis model was reconstructed to examine changes in effect estimates and credible intervals for each treatment. This analysis was used to assess the influence of individual studies on the overall results. If exclusion of any single study did not materially change the direction of effect or the statistical interpretation, the findings were considered robust.

### 2.13 Publication bias assessment

Contour-enhanced funnel plots and Egger’s regression test were used to assess potential publication bias or small-study effects for the main efficacy outcomes (18). The outcomes analyzed included PASI90, PASI75, PASI100, and IGA/PGA/sPGA 0/1. If the funnel plot was generally symmetrical and the P value of Egger’s test was greater than 0.05, no obvious evidence of publication bias was considered to be present; however, these tests were interpreted cautiously because the number of studies was limited.

### 2.14 Certainty of evidence assessment

The certainty of evidence was assessed using the GRADE framework in GRADEpro GDT. In the GRADE approach, evidence from randomized controlled trials starts as high certainty and may be rated down across five domains: risk of bias, inconsistency, indirectness, imprecision, and publication bias. Certainty was summarized as high, moderate, low, or very low, reflecting confidence in the comparison-specific effect estimate rather than the magnitude or statistical significance of the effect. In the revised profiles, certainty was retained as high for robust placebo-controlled PASI75 and IGA/PGA/sPGA 0/1 outcomes and rated as moderate where imprecision or indirectness was the main concern; no outcome was rated below moderate.

## 3 Results

### 3.1 Literature search results

**Figure 1:**
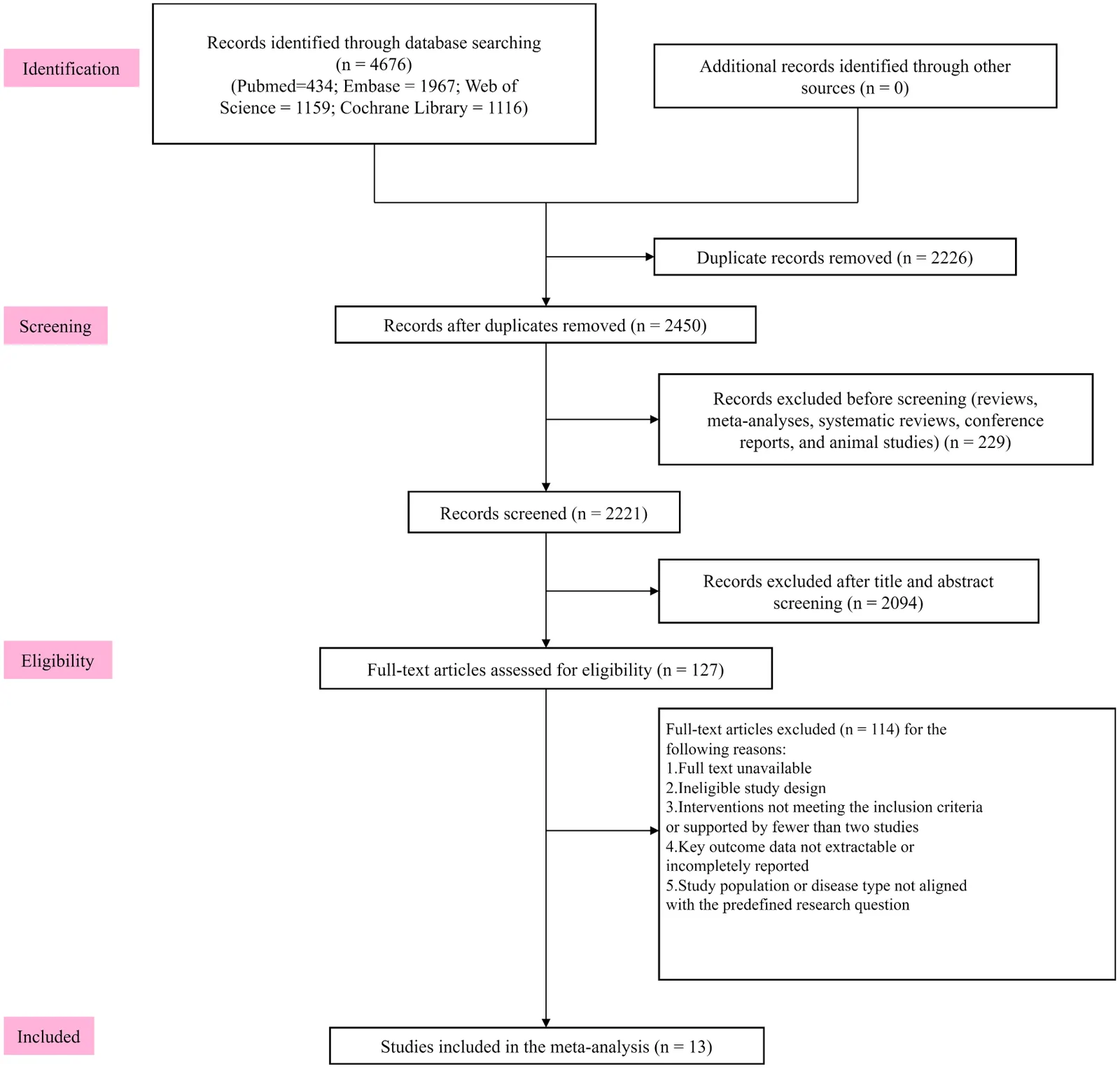
Literature screening flow diagram.

The initial database search identified 4, 676 records, including 434 from PubMed, 1, 967 from Embase, 1, 159 from Web of Science, and 1, 116 from the Cochrane Library; no additional records were identified from other sources. After removal of duplicates, 2, 450 records remained. A further 229 records, including reviews, meta-analyses, systematic reviews, conference abstracts, and animal studies that clearly did not meet the inclusion criteria, were excluded, leaving 2, 221 records for title and abstract screening. After title and abstract screening, 2, 094 records were excluded, and 127 articles were assessed in full text. After full-text review, 114 articles were excluded because the full text was unavailable, the study design did not meet the eligibility criteria, the intervention was inconsistent with the predefined criteria, fewer than two eligible studies were available for the relevant drug, key outcome data were unavailable or incompletely reported, or the study population or disease type did not match the prespecified research question. Finally, 13 articles were included in the systematic review and network meta-analysis. Notably, two articles each contained two independent RCTs; therefore, risk-of-bias assessment and some subsequent analyses were performed at the level of independent RCTs. The literature screening process is shown in **Figure 1**.

### 3.2 Basic characteristics of included studies

A total of 13 articles, comprising 15 RCTs or independent study units, were included. The UltIMMa study included two RCTs, UltIMMa-1 and UltIMMa-2, and the reSURFACE study included two RCTs, reSURFACE 1 and reSURFACE 2; these trials were therefore treated as independent study units in the analysis. All included studies enrolled patients with plaque psoriasis. The study regions covered North America, Europe, and Asia, and most studies were international multicenter clinical trials.

The included studies evaluated four IL-23 p19 monoclonal antibodies: guselkumab, risankizumab, tildrakizumab, and mirikizumab. The base-case control groups received placebo. The main efficacy outcomes included PASI75, PASI90, PASI100, and IGA/PGA/sPGA 0/1; safety outcomes included AE and SAE. Most studies reported baseline age and sex distribution, whereas additional baseline covariates were not sufficiently complete for a full arm-level quantitative presentation across all treatment nodes. For multi-dose studies, only the target dose arm included in the network meta-analysis was extracted. If baseline data for the target dose arm were not reported separately in the original article, pooled treatment-arm baseline data were used for description. The basic characteristics of the included studies are presented in **Table 1**.

**Table 1:** Basic characteristics of included studies.

No.	Included study / trial unit	Study region	Participants (T/C)	Mean age (T/C)	Sex: male/female (T/C)	Intervention (T)	Comparator (C)	Main outcomes
1	Kenneth B. Gordon—2015 (19)	North America and Europe; 43 centers	42/42	44.0/46.5	149/59 (pooled GUS arms) / 28/14	Guselkumab 100 mg	Placebo	Week 16 PGA 0/1, PASI75, PASI90, PASI100; AE, SAE
2	Andrew Blauvelt—2017, VOYAGE 1 (9)	International multicenter; 101 centers	329/174	43.9 ± 12.74 / 44.9 ± 12.90	240/89 / 119/55	Guselkumab 100 mg	Placebo	Week 16 IGA 0/1, PASI90, PASI75, PASI100; AE, SAE
3	Kristian Reich—2017, VOYAGE 2 (20)	International multicenter; 115 centers	496/248	43.7 ± 12.2 / 43.3 ± 12.4	349/147 / 173/75	Guselkumab 100 mg	Placebo	Week 16 IGA 0/1, PASI90, PASI75, PASI100; AE, SAE
4	O. Nemoto—2018 (21)	Japan; single center	5/4	Not separately reported for the selected dose	15/5 (pooled GUS arms) / 4/1	Guselkumab 100 mg	Placebo	Week 16 PASI, PGA, safety, pharmacokinetics, immunogenicity
5	Kenneth B. Gordon—2018, UltIMMa-1 (8)	Multinational; 139 centers	304/102	48.3 ± 13.4 / 49.3 ± 13.6	212/92 / 79/23	Risankizumab 150 mg	Placebo	Week 16 PASI90, sPGA 0/1, PASI75, PASI100; AE, SAE
6	Kenneth B. Gordon—2018, UltIMMa-2 (8)	Multinational; 139 centers	294/98	46.2 ± 13.7 / 48.6 ± 14.8	203/91 / 66/33	Risankizumab 150 mg	Placebo	Week 16 PASI90, sPGA 0/1, PASI75, PASI100; AE, SAE
7	Mamitaro Ohtsuki—2019, SustaIMM (22)	Japan	55/58	53.3 ± 11.9 / 50.9 ± 11.2	50/5 / 45/13	Risankizumab 150 mg	Placebo	Week 16 PASI90, PASI75, PASI100, sPGA 0/1; AE, SAE
8	Andrew Blauvelt—2020 (23)	Australia, Belgium, Canada, Czech Republic, France, Germany, Japan, Korea, USA; 60 centers	407/100	51 / 48	283/124 / 73/27	Risankizumab 150 mg	Placebo	Week 16 PASI90, sPGA 0/1, PASI75, PASI100; long-term maintenance outcomes; AE, SAE
9	K. Papp—2015 (24)	USA, Canada, Japan and Europe; 64 centers	89/46	43.2 ± 12.9 / 45.9 ± 11.7	31/11 (100 mg arm information incomplete) / 38/8	Tildrakizumab 100 mg	Placebo	Week 12 PASI75, PASI90, PGA clear/minimal, DLQI; AE, SAE
10	Kristian Reich—2017, reSURFACE 1 (25)	Australia, Canada, Japan, UK, USA	309/155	46.4 ± 13.1 / 47.9 ± 13.5	207/102 / 100/55	Tildrakizumab 100 mg	Placebo	Week 12 PASI75, PGA 0/1, PASI90, PASI100; AE, SAE
11	Kristian Reich—2017, reSURFACE 2 (25)	Europe, Israel and USA	307/156	44.6 ± 13.6 / 46.4 ± 12.2	220/87 / 112/44	Tildrakizumab 100 mg	Placebo	Week 12 PASI75, PGA response, PASI90, PASI100; AE, SAE
12	Chen Yu—2024 (26)	China	110/110	39.0 (18.0–66.0) / 41.0 (19.0–67.0)	82/28 / 85/25	Tildrakizumab 100 mg	Placebo	Week 12 PASI75, PGA response, PASI90, PASI100; AE, SAE
13	Kim Papp—2023, OASIS-2 (27)	Multinational; 178 centers	905/112	45.8 ± 13.0 / 43.8 ± 12.5	607/298 / 82/30	Mirikizumab 250 mg	Placebo	Week 16 sPGA 0/1, PASI90, PASI75, PASI100; week 52 long-term outcomes; AE, SAE
14	Andrew Blauvelt—2022, OASIS-1 (28)	Germany, Japan, Korea, Mexico, Poland, Russian Federation, Taiwan, USA and Puerto Rico; 69 centers	423/107	46.4 ± 13.6 / 45.7 ± 13.7	299/124 / 74/33	Mirikizumab 250 mg	Placebo	Week 16 sPGA 0/1, PASI90, PASI75, PASI100; maintenance outcomes; AE, SAE
15	Kristian Reich—2019 (29)	Canada, Germany, Japan, Poland and USA; 40 centers	51/52	49.2 ± 13.3 / 46.0 ± 12.4	39/12 / 42/10	Mirikizumab 300 mg	Placebo	Week 16 PASI90, PASI75, PASI100, sPGA 0/1, BSA ≤ 1%, PSSI, PSS, DLQI; AE, SAE

T, treatment group; C, placebo group; PASI, Psoriasis Area and Severity Index; PGA/IGA/sPGA, Physician/Investigator/static Physician Global Assessment; AE, adverse event; SAE, serious adverse event. In multi-dose studies, participant numbers were extracted for the dose arm included in the network meta-analysis. When age or sex was not reported separately for the selected dose arm, the pooled treatment-arm baseline data reported in the original article were used and noted in the table.

### 3.3 Risk-of-bias assessment

**Figure 2:**
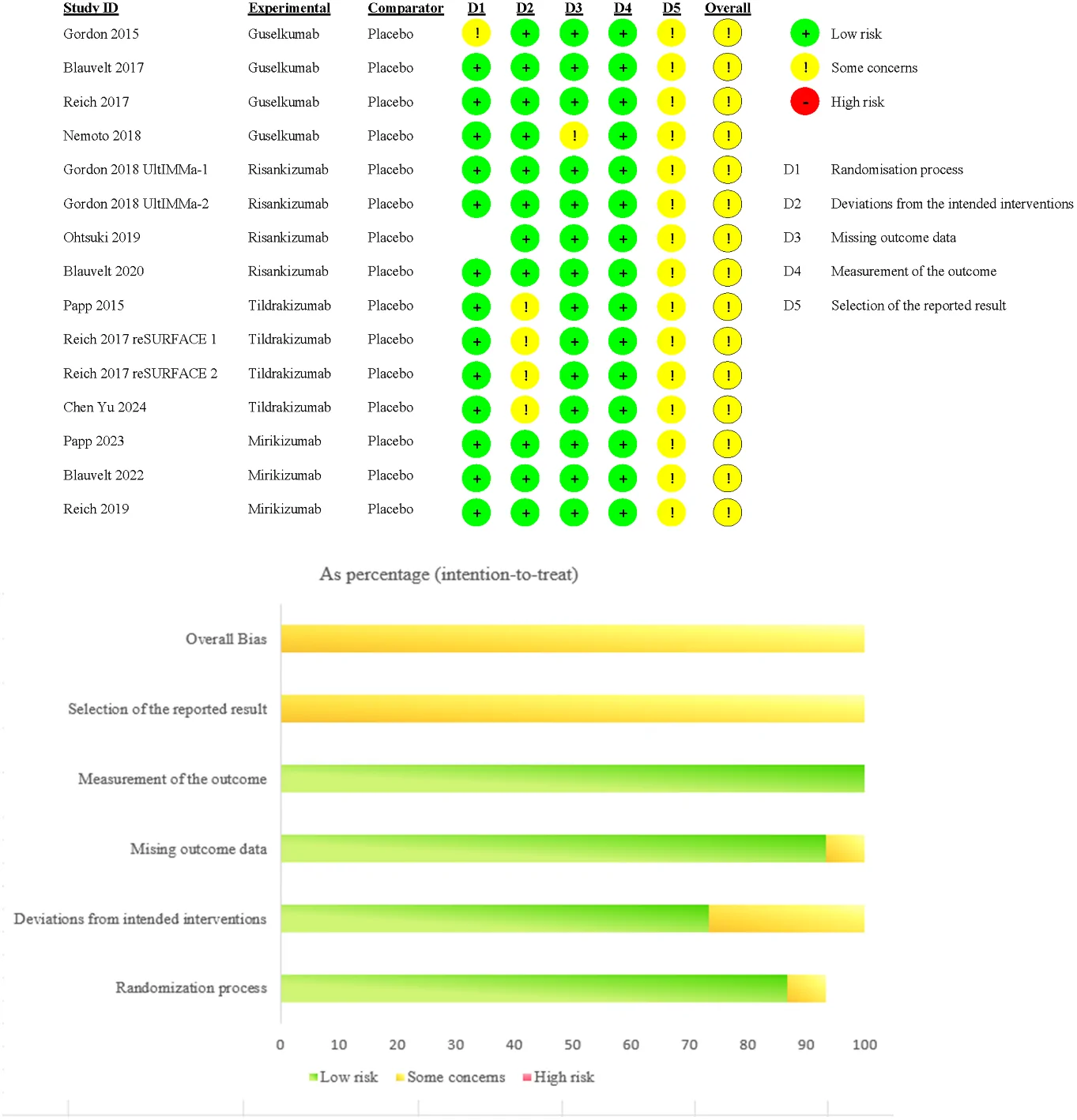
Risk of bias assessment of included studies.

The RoB 2.0 tool was used to assess the risk of bias in the included RCTs. Overall, the methodological quality of the included studies was relatively stable, and no study was judged to have an overall high risk of bias. However, most studies were rated as having “some concerns” in the overall assessment. The main source of bias was selection of the reported result, namely domain D5, suggesting some uncertainty regarding prespecified outcomes, analysis plans, or completeness of reporting in some studies. Some concerns were also identified in a small number of studies regarding the randomization process, domain D1; deviations from intended interventions, domain D2; and missing outcome data, domain D3. In contrast, the risk of bias in measurement of the outcome, domain D4, was generally low. Overall, the included studies did not show an evident systematic high risk of bias, but potential selective reporting should still be considered when interpreting the certainty of evidence. The risk-of-bias assessment is shown in **Figure 2**.

### 3.4 Evidence network structure

Evidence networks were constructed separately for six outcomes: PASI90, PASI75, PASI100, IGA/PGA/sPGA, AE, and SAE. The overall network structures were generally consistent across outcomes, with placebo as the central comparator. Guselkumab, risankizumab, tildrakizumab, and mirikizumab each formed direct comparisons with placebo. No direct head-to-head comparisons were observed among different IL-23 p19 monoclonal antibodies; therefore, the relative efficacy between active agents was mainly derived from indirect evidence using placebo as the common comparator.

**Figure 3:**
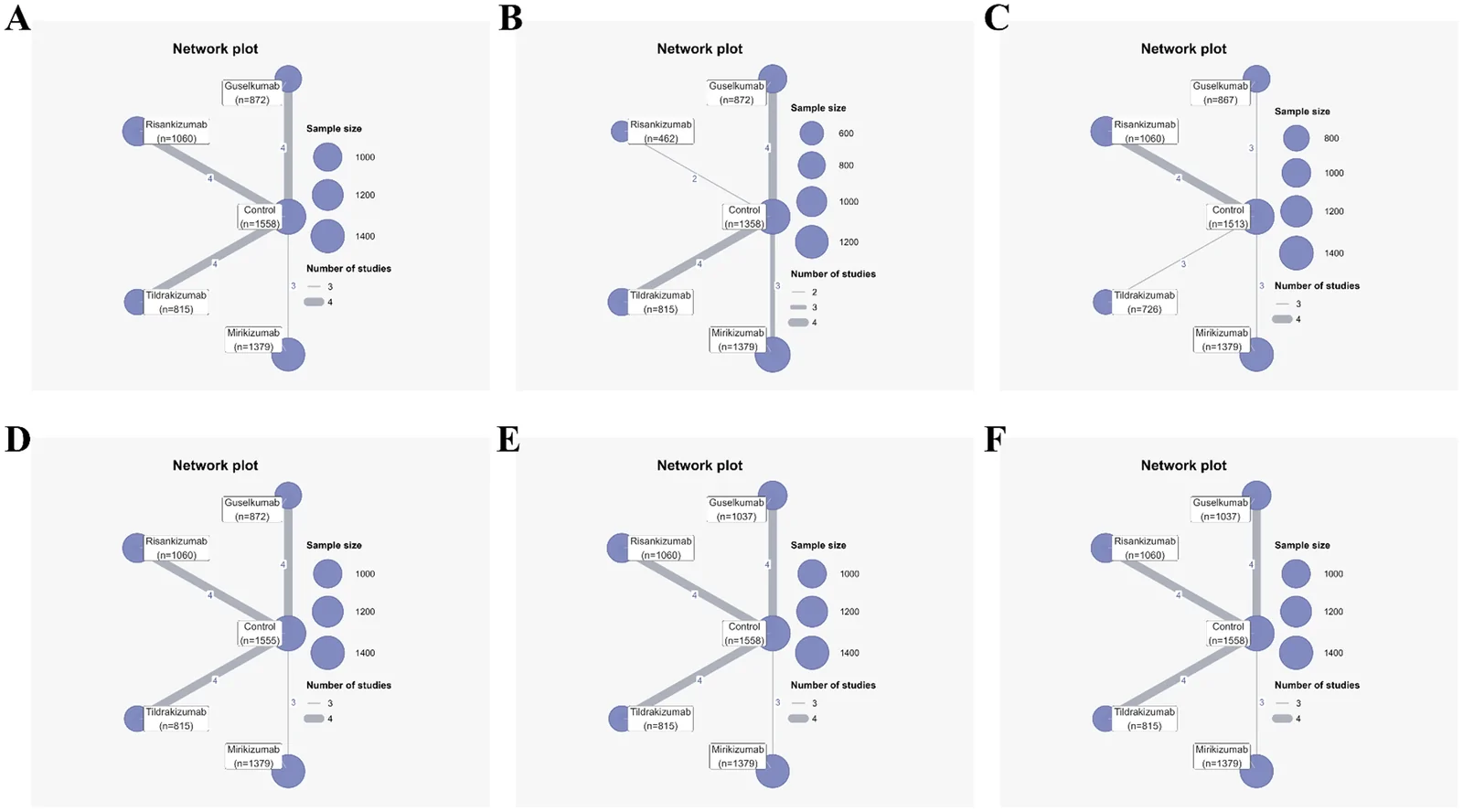
Network evidence plots of IL-23 p19 monoclonal antibodies for plaque psoriasis across different outcomes. **(A)** PASI90; **(B)** PASI75; **(C)** PASI100; **(D)** IGA/PGA/sPGA 0/1; **(E)** AE; **(F)** SAE. Each node represents an intervention, with node size proportional to the sample size included for that intervention. Lines between nodes indicate direct comparative evidence, and line thickness reflects the number of direct comparisons. PASI, Psoriasis Area and Severity Index; IGA, Investigator’s Global Assessment; PGA, Physician’s Global Assessment; sPGA, static Physician’s Global Assessment; AE, adverse event; SAE, serious adverse event.

For the PASI90 outcome, the network included placebo, guselkumab, risankizumab, tildrakizumab, and mirikizumab. The sample sizes were 1, 558 for placebo, 1, 060 for risankizumab, 872 for guselkumab, 815 for tildrakizumab, and 1, 379 for mirikizumab. The network structures for PASI75, PASI100, IGA/PGA/sPGA, and safety outcomes were generally similar to that for PASI90, although the sample size of each node varied slightly across outcomes. Overall, the evidence network was primarily based on direct comparisons between each drug and placebo, indicating that this study was well suited to evaluate the efficacy and safety of each drug versus placebo. However, uncertainty should be considered when interpreting indirect comparisons between active agents. The evidence networks are shown in **Figure 3**.

### 3.5 Assessment of transitivity

**Figure 4:**
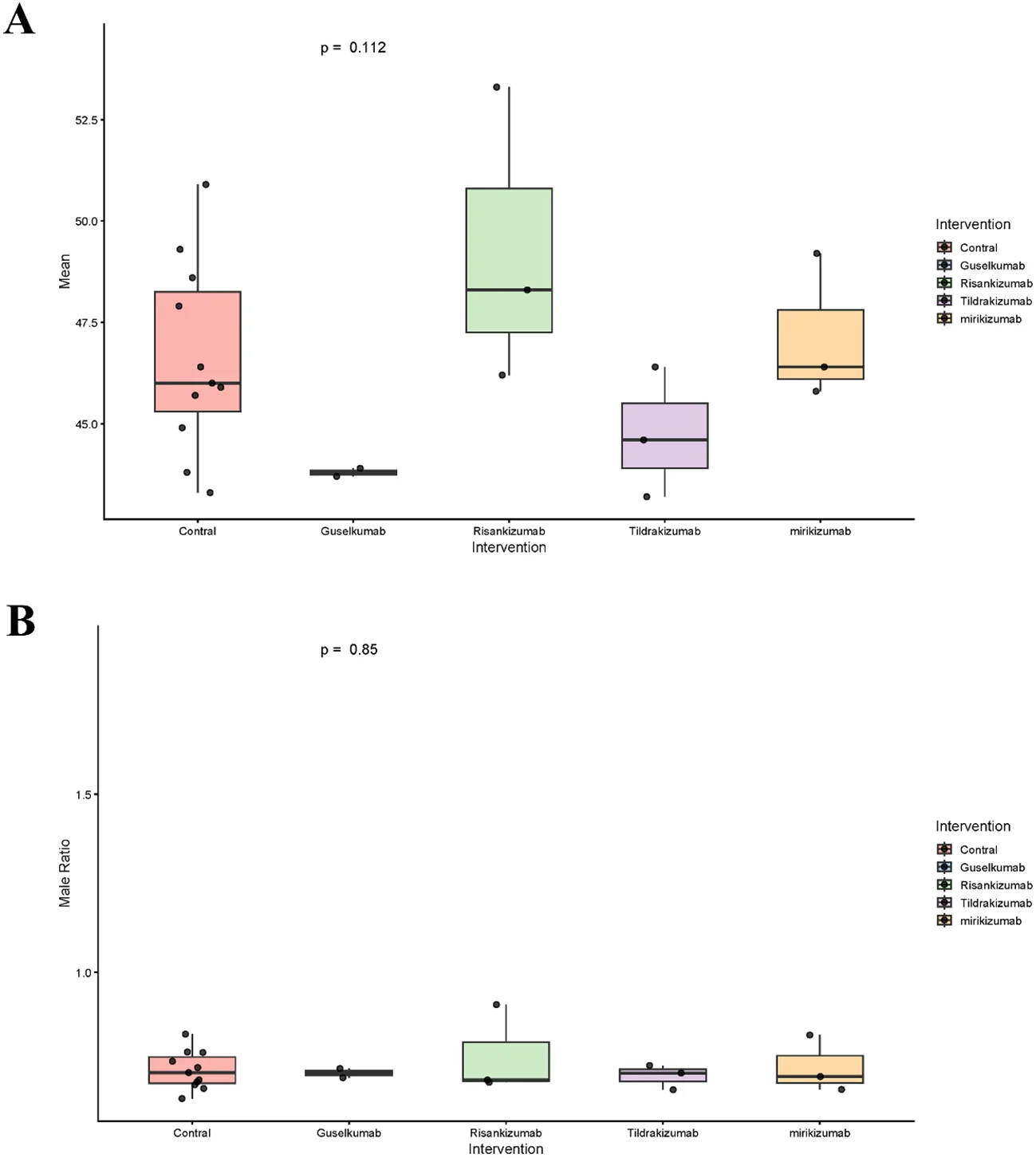
Assessment of transitivity across treatment groups. **(A)** distribution of mean age across treatment groups; **(B)** distribution of male proportion across treatment groups. Key baseline characteristics were compared across interventions to assess the transitivity assumption of the network meta-analysis. The distributions of age and sex were generally balanced across treatment groups, with no evident systematic differences.

To assess whether the transitivity assumption of the network meta-analysis was satisfied, differences in key baseline characteristics across treatment groups were evaluated using mean age and the proportion of male participants. The results showed that mean age was generally comparable across treatment groups, with no statistically significant between-group difference (P = 0.112). The proportion of male participants was also balanced across treatment groups, with no statistically significant difference (P = 0.85). These findings support the basic transitivity assumption for age and sex. Other potentially relevant effect modifiers were incompletely and inconsistently reported and therefore could not be fully assessed quantitatively. Therefore, the transitivity assumption should be considered broadly plausible but not definitively proven. The assessment of transitivity is shown in **Figure 4**.

### 3.6 Heterogeneity analysis

**Figure 5:**
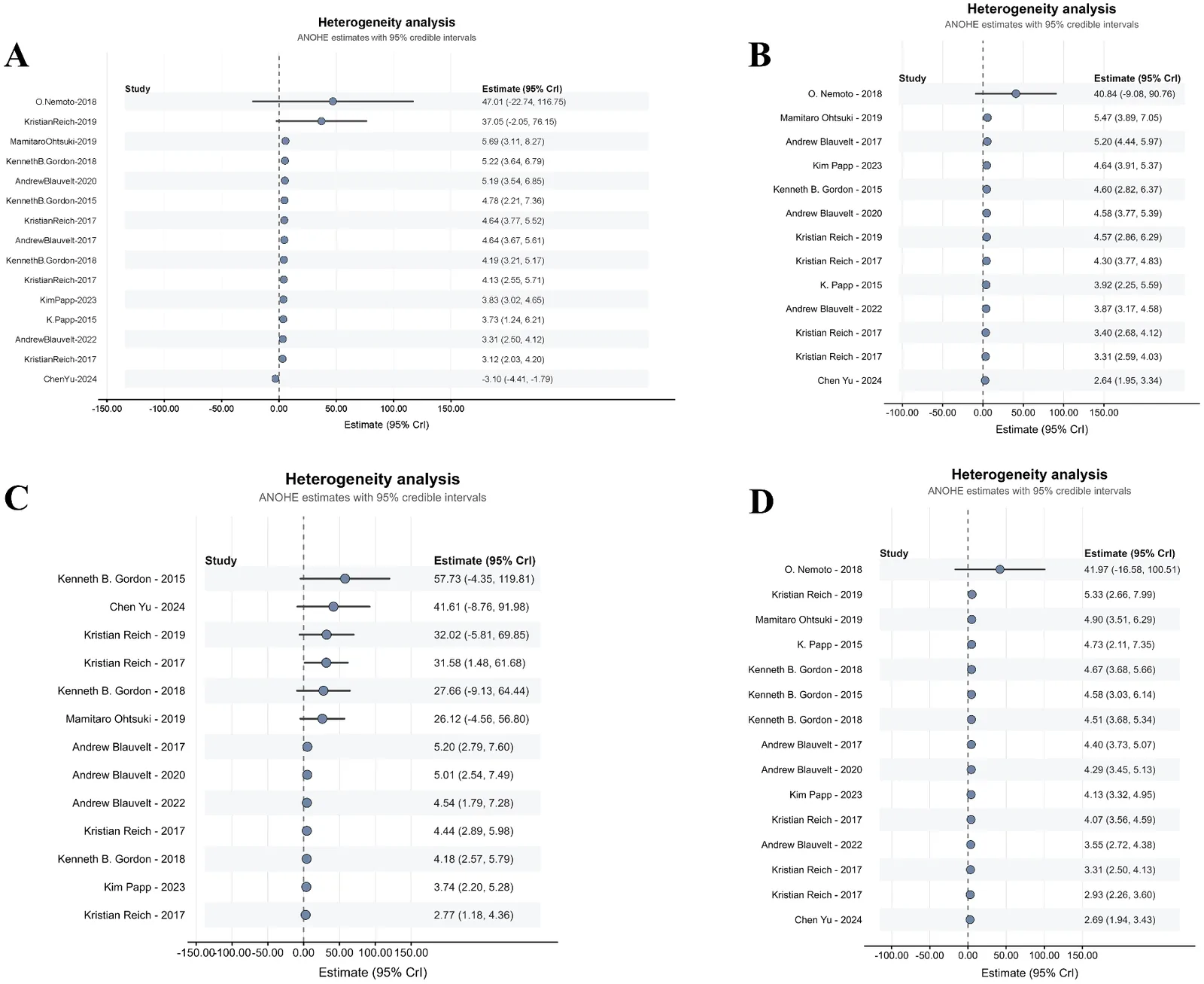
Heterogeneity analysis across major efficacy outcomes. **(A)** PASI90; **(B)** PASI75; **(C)** PASI100; **(D)** IGA/PGA/sPGA 0/1. The figure shows the effect estimates and uncertainty intervals of individual studies for each efficacy outcome, allowing assessment of between-study heterogeneity and the potential influence of individual studies on the overall network estimates.

Heterogeneity analysis was further performed for four major efficacy outcomes: PASI90, PASI75, PASI100, and IGA/PGA/sPGA. The results showed that the contribution of individual studies to overall heterogeneity varied across outcomes. For PASI90, studies such as O. Nemoto-2018 and Kristian Reich-2019 had relatively wide estimation intervals, suggesting that they may have contributed substantially to the uncertainty of this outcome. For PASI75, the effect estimate and credible interval of O. Nemoto-2018 were relatively prominent, although most studies showed relatively concentrated estimates. For PASI100, studies such as Kenneth B. Gordon-2015, Chen Yu-2024, and Kristian Reich-2019 had wide estimation intervals, indicating some between-study variability for this outcome. For IGA/PGA/sPGA, most study estimates were relatively concentrated, with only a few studies showing wider credible intervals. Overall, none of the four efficacy outcomes appeared to be driven by a single study with abnormal heterogeneity, although some between-study variability indicated that the results should be interpreted in conjunction with sensitivity analyses. The heterogeneity analysis is shown in **Figure 5**.

### 3.7 Efficacy and safety of IL-23 p19 monoclonal antibodies compared with placebo

Network meta-analysis was performed using the placebo group as the reference. For efficacy outcomes, an OR greater than 1 indicated a higher response rate for IL-23 p19 monoclonal antibodies compared with placebo.

For PASI90, risankizumab, guselkumab, and mirikizumab were all significantly superior to placebo, with ORs of 146.88 (95% CrI: 12.07–1866.23), 121.10 (95% CrI: 8.41–2093.11), and 97.18 (95% CrI: 5.59–2376.62), respectively. Tildrakizumab also showed a trend toward benefit compared with placebo, but the 95% CrI crossed the null value (OR = 6.41, 95% CrI: 0.55–78.88), indicating that its advantage for this outcome was not stable. For PASI75, all IL-23 p19 monoclonal antibodies were significantly superior to placebo. The ORs were 124.04 (95% CrI: 48.40–347.64) for risankizumab, 105.41 (95% CrI: 53.36–219.34) for guselkumab, 72.36 (95% CrI: 35.70–153.19) for mirikizumab, and 24.05 (95% CrI: 13.45–45.67) for tildrakizumab. These findings indicate that all four IL-23 p19 monoclonal antibodies significantly improved PASI75 response rates. For PASI100, all four drugs were significantly superior to placebo. The ORs were 145.14 (95% CrI: 32.60–1548.07) for risankizumab, 122.53 (95% CrI: 24.49–1321.07) for guselkumab, 72.09 (95% CrI: 14.10–805.73) for mirikizumab, and 54.59 (95% CrI: 8.83–1605.44) for tildrakizumab. These results suggest that IL-23 p19 monoclonal antibodies provide significant advantages in achieving complete or near-complete skin clearance. For IGA/PGA/sPGA, all four drugs were also significantly superior to placebo. The ORs were 89.73 (95% CrI: 53.83–156.32) for risankizumab, 68.84 (95% CrI: 42.65–116.95) for guselkumab, 52.82 (95% CrI: 28.84–102.70) for mirikizumab, and 21.46 (95% CrI: 13.55–36.13) for tildrakizumab. These findings indicate that IL-23 p19 monoclonal antibodies significantly increased the proportion of patients achieving clear or almost clear physician global assessment outcomes.

**Figure 6:**
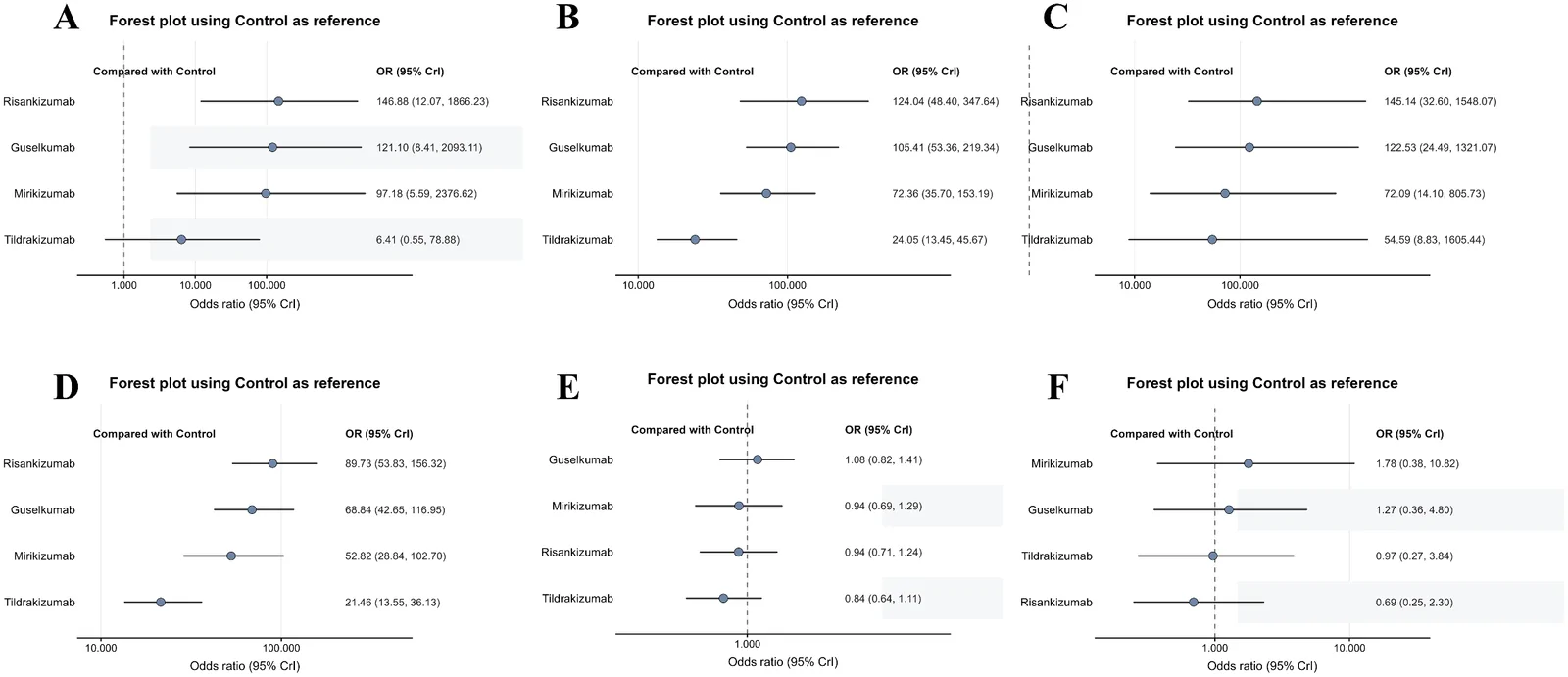
Forest plots of efficacy and safety comparing IL-23 p19 monoclonal antibodies with placebo. **(A)** PASI90; **(B)** PASI75; **(C)** PASI100; **(D)** IGA/PGA/sPGA 0/1; **(E)** AE; (F) SAE. Effect estimates are presented as odds ratios. For efficacy outcomes, an OR greater than 1 indicates a higher response rate for the IL-23 p19 monoclonal antibody compared with placebo. For safety outcomes, an OR greater than 1 indicates a higher risk of adverse events. Horizontal lines represent 95% credible intervals; intervals not crossing 1 indicate statistical significance. OR, odds ratio; CrI, credible interval.

Regarding safety, no significant differences were observed between any IL-23 p19 monoclonal antibody and placebo for overall AE. The ORs were 1.08 (95% CrI: 0.82-1.41) for guselkumab, 0.94 (95% CrI: 0.69-1.29) for mirikizumab, 0.94 (95% CrI: 0.71-1.24) for risankizumab, and 0.84 (95% CrI: 0.64-1.11) for tildrakizumab. Similarly, for SAE, no statistically significant differences were observed between any drug and placebo. The ORs were 1.78 (95% CrI: 0.38-10.82) for mirikizumab, 1.27 (95% CrI: 0.36-4.80) for guselkumab, 0.97 (95% CrI: 0.27-3.84) for tildrakizumab, and 0.69 (95% CrI: 0.25-2.30) for risankizumab. These findings suggest no clear increase in overall AE or SAE risk, but they should not be interpreted as excluding differences in specific adverse-event categories such as infections, serious infections, malignancy, injection-site reactions, or gastrointestinal disorders. The forest plots are shown in **Figure 6**, and full league tables are provided in **Supplementary Table 1**.

### 3.8 Relative efficacy comparisons among different IL-23 p19 monoclonal antibodies

Further analyses were conducted using different active drugs as references to compare the relative efficacy of IL-23 p19 monoclonal antibodies. These comparisons were mainly indirect because no direct head-to-head RCTs among the active IL-23 p19 agents were available. Overall, risankizumab, guselkumab, and mirikizumab showed similar effect estimates for most efficacy outcomes, and most pairwise comparisons among these three agents had 95% CrIs crossing the null value, suggesting no stable statistically significant differences among them.

**Figure 7:**
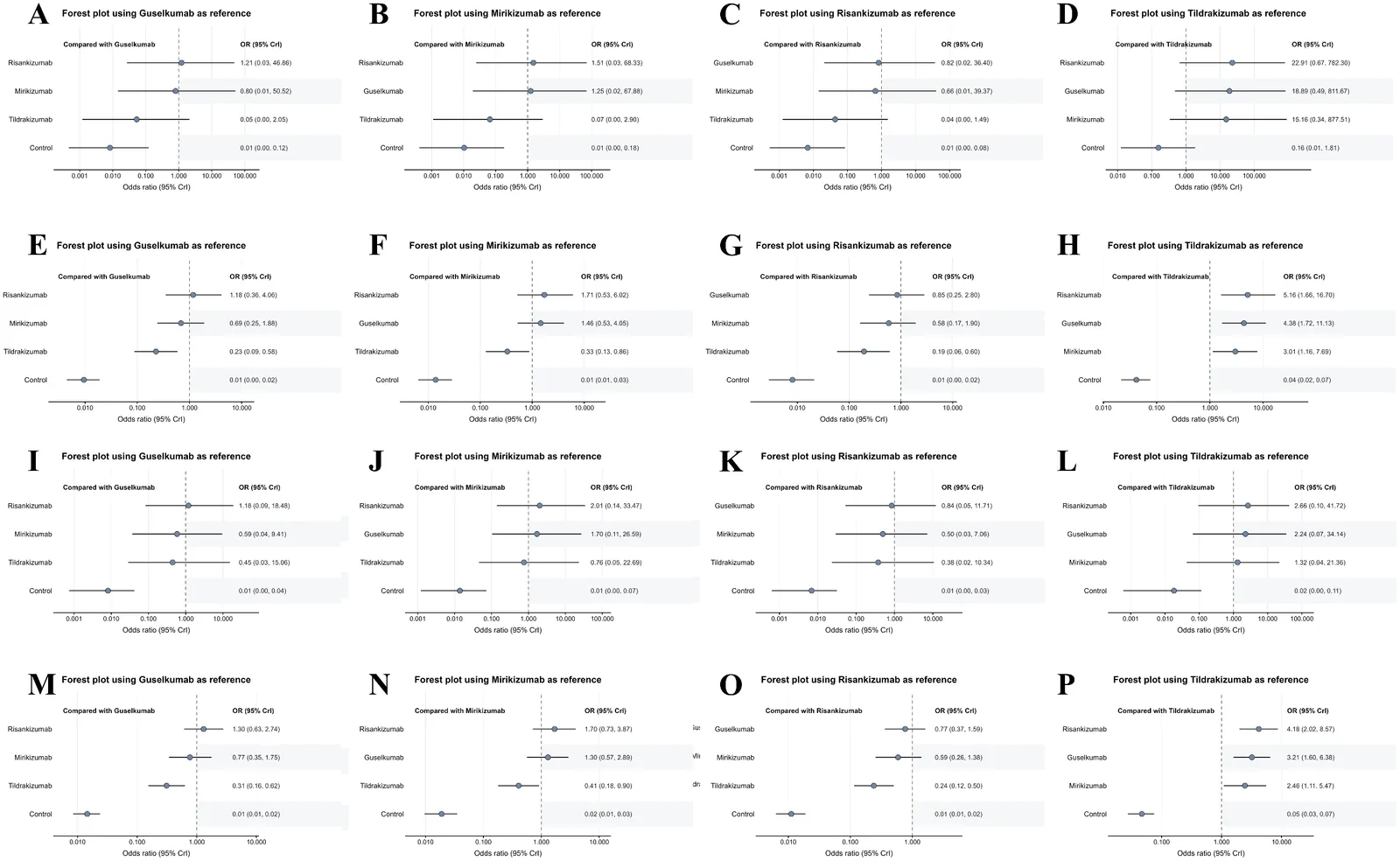
Forest plots of comparative efficacy among IL-23 p19 monoclonal antibodies using different active treatments as references. Panels (**A–D**) show PASI90, panels (**E–H**) show PASI75, panels (**I–L**) show PASI100, and panels (**M–P**) show IGA/PGA/sPGA 0/1. Within each outcome, guselkumab, mirikizumab, risankizumab, and tildrakizumab are used as the reference treatments in panels (**A, E, I, M**), (**B, F, J, N**), (**C, G, K, O**), and (**D, H, L, P**), respectively. Horizontal lines represent 95% credible intervals, and intervals not crossing 1 indicate statistical significance.

For PASI90, most pairwise comparisons among risankizumab, guselkumab, mirikizumab, and tildrakizumab did not reach statistical significance. Tildrakizumab generally showed lower effect estimates than the other agents, but the credible intervals were wide, indicating substantial uncertainty. For PASI75, tildrakizumab showed relatively weaker efficacy than the other three agents. When tildrakizumab was used as the reference, risankizumab, guselkumab, and mirikizumab had significantly higher PASI75 response rates, with ORs of 5.16 (95% CrI: 1.66–16.70), 4.38 (95% CrI: 1.72–11.13), and 3.01 (95% CrI: 1.16–7.69), respectively. No clear statistically significant differences were observed among risankizumab, guselkumab, and mirikizumab. For PASI100, no stable statistically significant differences were observed among the different IL-23 p19 monoclonal antibodies, suggesting that current evidence is insufficient to confirm that any one agent is significantly superior to the others in achieving complete skin clearance. For IGA/PGA/sPGA, tildrakizumab showed lower response rates than risankizumab, guselkumab, and mirikizumab. When tildrakizumab was used as the reference, the ORs were 4.18 (95% CrI: 2.02–8.57) for risankizumab, 3.21 (95% CrI: 1.60–6.38) for guselkumab, and 2.46 (95% CrI: 1.11–5.47) for mirikizumab. No stable significant differences were observed among risankizumab, guselkumab, and mirikizumab. Forest plots of pairwise comparisons among active drugs are shown in **Figure 7**.

### 3.9 SUCRA ranking analysis

To further evaluate the relative ranking of different IL-23 p19 monoclonal antibodies for efficacy outcomes, SUCRA cumulative ranking curves and rankograms were generated. Overall, risankizumab ranked first or near first across the four efficacy outcomes, followed by guselkumab, whereas mirikizumab showed intermediate performance and tildrakizumab ranked relatively lower. Placebo ranked lowest for all efficacy outcomes.

For PASI90, the SUCRA values in descending order were risankizumab, 77.8%; guselkumab, 73.7%; mirikizumab, 69.3%; tildrakizumab, 27.5%; and placebo, 1.6%. For PASI75, the ranking was risankizumab, 85.7%; guselkumab, 79.5%; mirikizumab, 59.2%; tildrakizumab, 25.6%; and placebo, 0.0%. For PASI100, the ranking was risankizumab, 77.0%; guselkumab, 72.1%; mirikizumab, 55.2%; tildrakizumab, 45.7%; and placebo, 0.0%. For IGA/PGA/sPGA, risankizumab had the highest SUCRA value, at 91.7%, followed by guselkumab, 74.4%; mirikizumab, 58.4%; tildrakizumab, 25.4%; and placebo, 0.0%.

**Figure 8:**
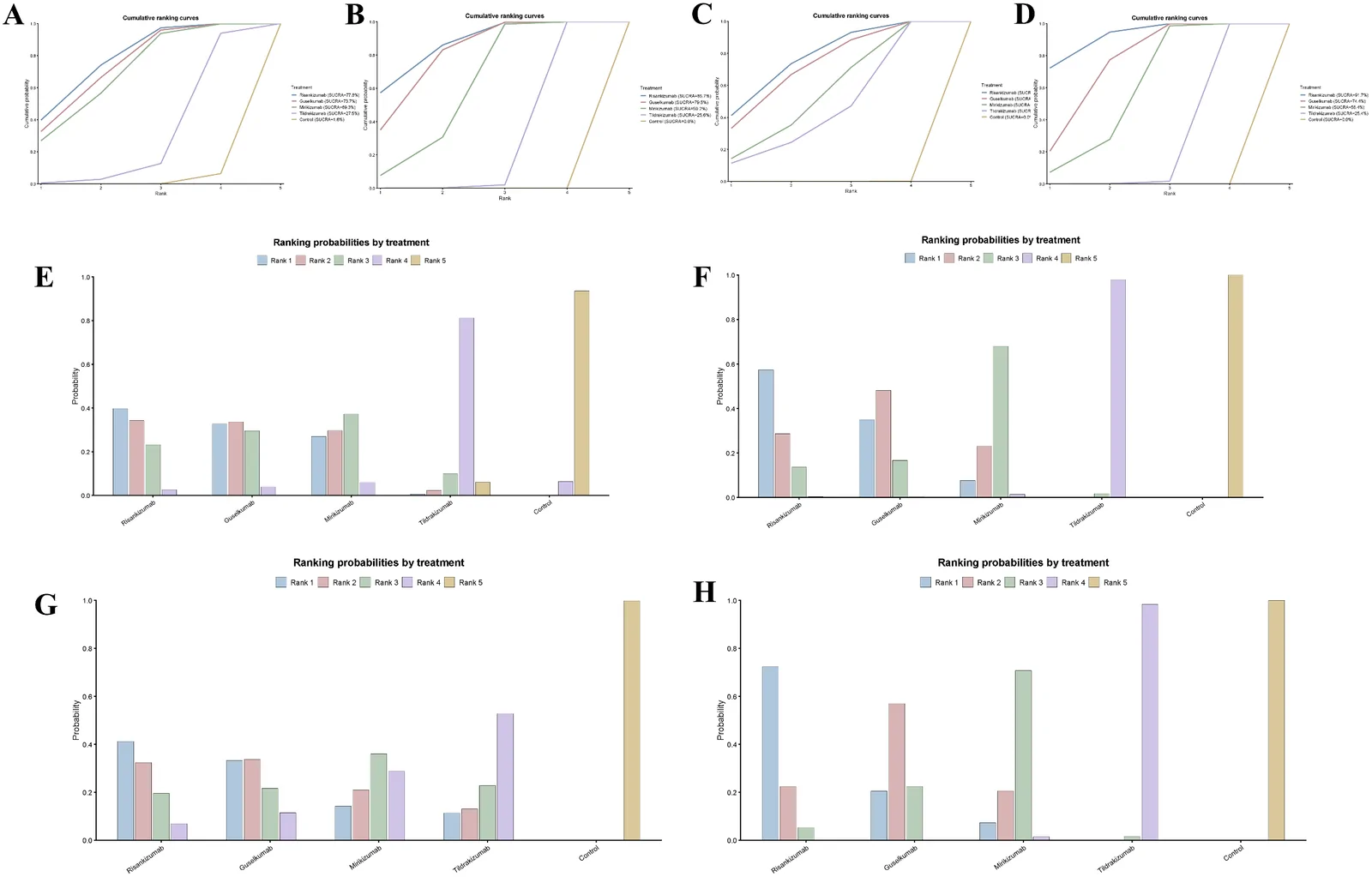
SUCRA ranking curves and rankograms of IL-23 p19 monoclonal antibodies for major efficacy outcomes. Panels (**A–D**) show cumulative ranking probability curves for PASI90, PASI75, PASI100, and IGA/PGA/sPGA 0/1, respectively. Panels (**E–H**) show the corresponding rankograms for PASI90, PASI75, PASI100, and IGA/PGA/sPGA 0/1, respectively. Higher SUCRA values indicate a better overall ranking for the corresponding outcome.

These results suggest that risankizumab had a high cumulative probability of ranking favorably across the four core efficacy outcomes, including PASI90, PASI75, PASI100, and IGA/PGA/sPGA. However, because SUCRA rankings do not by themselves establish statistically significant superiority and most active-agent comparisons among risankizumab, guselkumab, and mirikizumab had overlapping 95% CrIs, the ranking results should be viewed as exploratory and hypothesis-generating. Guselkumab also showed stable efficacy advantages, mirikizumab showed intermediate efficacy, and tildrakizumab, although significantly superior to placebo for several outcomes, ranked relatively lower in indirect comparisons and ranking analyses. The ranking curves and rankograms are shown in **Figure 8**.

### 3.10 Sensitivity analysis

**Figure 9:**
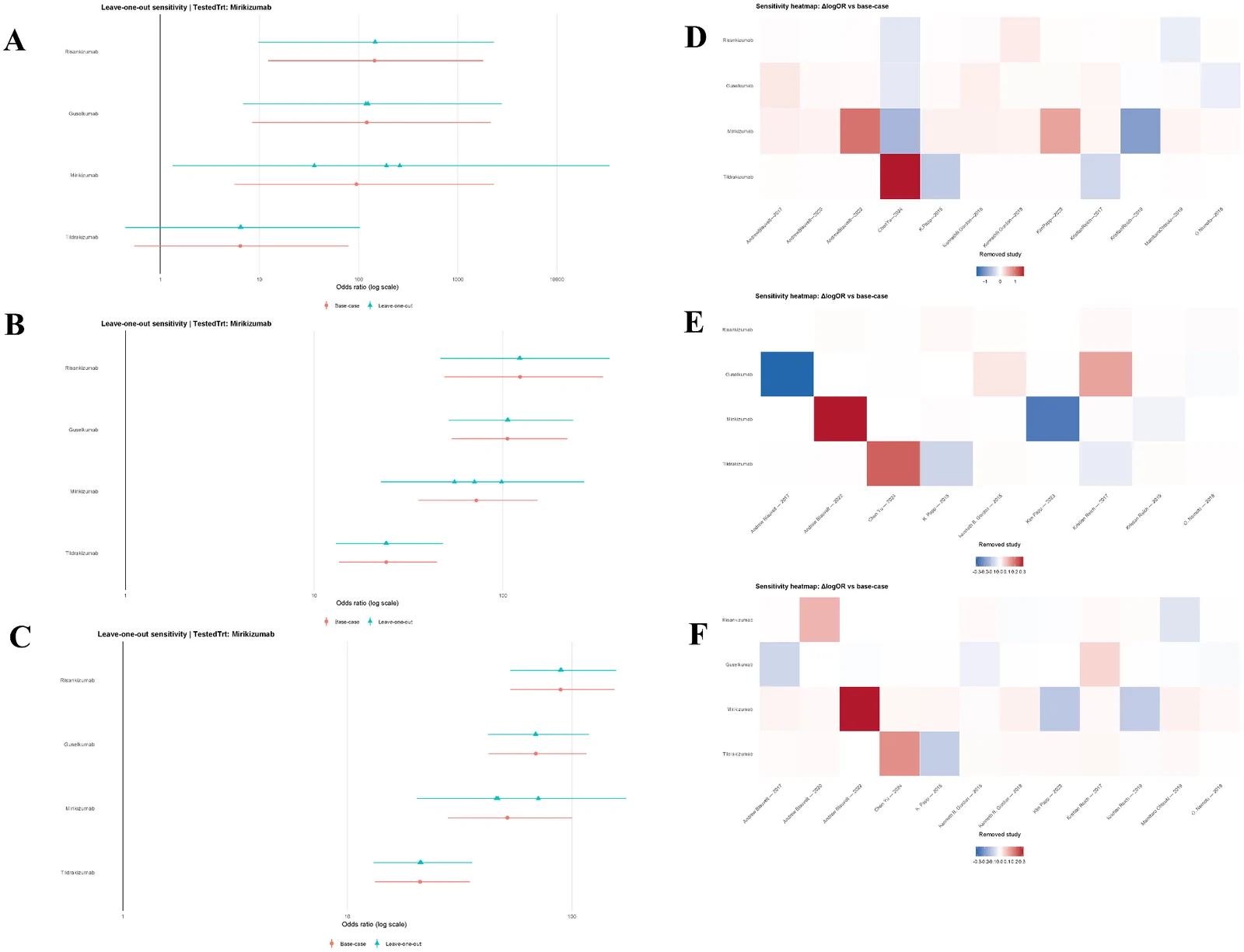
Leave-one-out sensitivity analysis for three major efficacy outcomes. Panels (**A–C**) show leave-one-out forest plots for PASI90, PASI75, and IGA/PGA/sPGA 0/1, respectively. Panels (**D–F**) show the corresponding heatmaps of changes in log odds ratios compared with the base-case model for PASI90, PASI75, and IGA/PGA/sPGA 0/1, respectively. Each study was sequentially excluded and the network meta-analysis model was refitted. Stronger color intensity indicates a larger change after exclusion of the corresponding study. This analysis evaluates whether the direction and interpretation of the main estimates are driven by any single study.

Leave-one-out sensitivity analyses were performed for four efficacy outcomes: PASI90, PASI75, PASI100, and IGA/PGA/sPGA. Each study was sequentially removed, the network model was refitted, and changes in treatment effects relative to the base-case estimates were evaluated. The heatmaps indicated that exclusion of some individual studies affected the magnitude of effect estimates for certain drugs, particularly in outcomes with smaller sample sizes or wider CrIs. However, the overall direction of effect remained consistent, and no result showed a clear reversal in direction. These findings suggest that the main network estimates were not highly dependent on any single study, although numerical uncertainty remained for sparse outcomes. The sensitivity analysis results are shown in **Figure 9**.

### 3.11 Publication bias assessment

**Figure 10:**
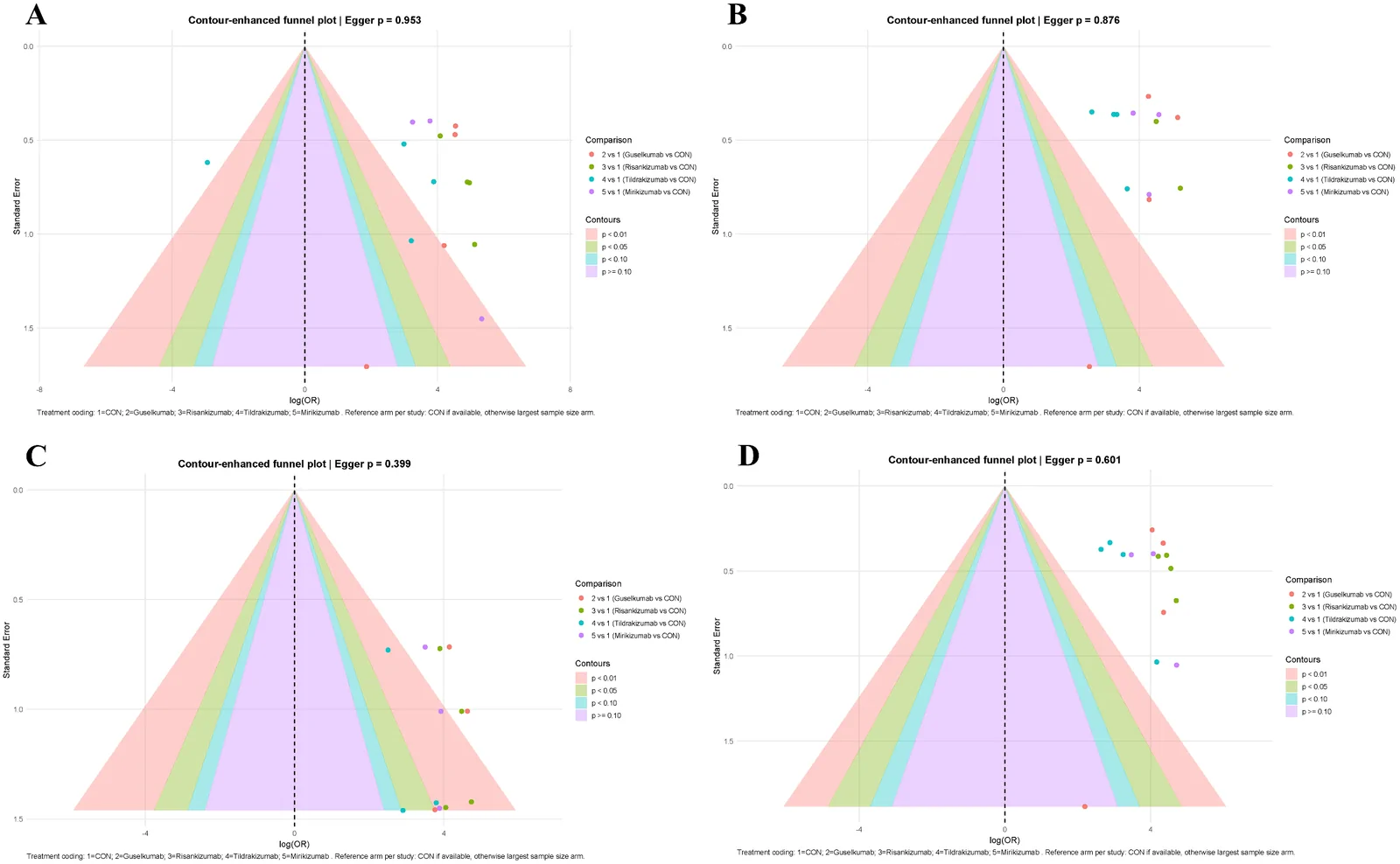
Publication bias assessment for major efficacy outcomes. **(A)** PASI90; **(B)** PASI75; **(C)** PASI100; **(D)** IGA/PGA/sPGA 0/1. Contour-enhanced funnel plots and Egger’s regression tests were used to evaluate potential publication bias or small-study effects. Each point represents a study or comparison. A more symmetrical funnel plot suggests a lower likelihood of publication bias. An Egger’s test P value greater than 0.05 indicates no statistically significant evidence of publication bias.

Contour-enhanced funnel plots and Egger’s tests were used to assess potential publication bias or small-study effects for PASI90, PASI75, PASI100, and IGA/PGA/sPGA. The results showed that the P values of Egger’s tests were 0.953 for PASI90, 0.876 for PASI75, 0.399 for PASI100, and 0.601 for IGA/PGA/sPGA, none of which reached statistical significance. Although the study points in the funnel plots showed some directional distribution because most comparisons used placebo as the common comparator, no clear evidence of small-study effects or publication bias was observed. Given the limited number of studies included for each outcome, however, the statistical power of publication bias assessment may be insufficient, and these results should therefore be interpreted with caution. The publication bias assessment is shown in **Figure 10**.

### 3.12 Summary of safety results

The safety network meta-analysis showed no significant differences between any of the four IL-23 p19 monoclonal antibodies and placebo in overall AE or SAE. For AE, the ORs of all drugs were close to 1, and all 95% CrIs crossed the null value. For SAE, although the point estimates for mirikizumab and guselkumab were slightly above 1 and that for risankizumab was below 1, the CrIs were wide and crossed the null value for all drugs, indicating imprecision due to sparse serious events. Overall, current evidence does not suggest a clear increase in overall AE or SAE risk, but this analysis cannot determine whether individual adverse-event categories differ among agents.

### 3.13 Certainty of evidence

The GRADEpro GDT evidence profiles indicated high certainty for the more robust placebo-controlled PASI75 and IGA/PGA/sPGA 0/1 comparisons. Certainty was rated as moderate for PASI90 and PASI100 because these higher-threshold efficacy outcomes had wider CrIs in some comparisons, and for AE and SAE because safety estimates, particularly serious events, were affected by sparse events and imprecision. Active-agent comparisons were also rated as moderate because they were primarily indirect comparisons through placebo rather than direct head-to-head RCT evidence. The complete GRADE evidence profiles are provided in the separate Supplementary GRADE Evidence File.

## 4 Discussion

Based on 13 articles comprising 15 RCTs, this study systematically evaluated the clinical benefits and safety of four IL-23 p19 monoclonal antibodies in moderate-to-severe plaque psoriasis. Overall, guselkumab, risankizumab, tildrakizumab, and mirikizumab showed clear advantages over placebo in efficacy outcomes, including PASI75, PASI90, PASI100, and IGA/PGA/sPGA 0/1. Regarding safety, none of the agents showed a significantly increased risk of overall AE or SAE compared with placebo, although specific adverse-event categories were not synthesized. Ranking analysis suggested that risankizumab had the highest probability of favorable efficacy across several outcomes, followed by guselkumab and mirikizumab; however, this ranking should be interpreted in the context of indirect comparisons, overlapping CrIs, and certainty of evidence.

The findings of this study are highly consistent with the immunopathogenesis of psoriasis. The IL-23/Th17 axis is considered a central pathway in the development and maintenance of chronic inflammation in psoriasis. IL-23 promotes and sustains pathogenic Th17-cell responses, thereby inducing the expression of inflammatory cytokines such as IL-17 and IL-22, which contribute to abnormal keratinocyte proliferation, inflammatory cell infiltration, and epidermal barrier dysfunction (5). Previous mechanistic studies and reviews have shown that IL-23, IL-17, and TNF-α jointly participate in the inflammatory network of psoriasis, with IL-23 playing a critical role in maintaining pathogenic Th17 cells (30–32). Therefore, selective blockade of the IL-23 p19 subunit can suppress upstream Th17-mediated inflammation and reduce downstream inflammatory amplification, which mechanistically explains the significant efficacy of IL-23 p19 monoclonal antibodies in outcomes such as PASI75, PASI90, and PASI100. Unlike blockade of the shared p40 subunit of IL-12/23, selective inhibition of the p19 subunit more specifically targets the IL-23 pathway while relatively preserving IL-12-related immune function, which may partly explain why this drug class combines strong efficacy with favorable safety (33).

From the perspective of clinical endpoints, this study included not only PASI75 but also more stringent outcomes such as PASI90, PASI100, and IGA/PGA/sPGA 0/1, which better reflects the evolving treatment goals for psoriasis. The treat-to-target concept has increasingly emphasized PASI90 or an absolute PASI score ≤3 as important indicators of clinical remission, while highlighting that skin clearance, quality-of-life improvement, and long-term safety are all central components of modern psoriasis management (34). In the present study, IL-23 p19 monoclonal antibodies showed significant advantages over placebo in PASI90, PASI100, and physician global assessment outcomes indicating clear or almost clear skin, suggesting that this drug class can not only achieve conventional clinical improvement but also meet the contemporary demand for higher levels of skin clearance.

This study found that risankizumab ranked highest across multiple efficacy outcomes, guselkumab also showed stable efficacy, whereas tildrakizumab ranked relatively lower. This pattern is broadly consistent with the overall findings of pivotal phase III trials and indirect comparative studies. The VOYAGE studies showed that guselkumab significantly improved IGA 0/1 and PASI90 response rates at week 16 compared with placebo (35, 36). The UltIMMa-1 and UltIMMa-2 trials demonstrated that risankizumab significantly improved PASI90 and sPGA 0/1 responses at week 16 compared with both placebo and ustekinumab (37, 38). The reSURFACE 1 and reSURFACE 2 trials showed that tildrakizumab had clear efficacy and good tolerability compared with placebo (39, 40). The OASIS studies and one phase II trial provided three mirikizumab RCTs for plaque psoriasis in this network (27–29). It should be noted that mirikizumab is less established for plaque psoriasis than guselkumab, risankizumab, and tildrakizumab; current FDA and EMA product information focused on inflammatory bowel disease indications rather than plaque psoriasis (41, 42). Therefore, mirikizumab findings should be interpreted as trial-based evidence within this NMA rather than as evidence of the same regulatory positioning as established psoriasis agents. More importantly, the inter-drug comparisons in this study were mainly based on indirect evidence using placebo as the common comparator rather than on sufficient head-to-head RCT evidence. Treatment rankings should therefore be interpreted as probabilistic estimates rather than definitive superiority or inferiority.

Safety is a key consideration in long-term biologic therapy. The present study showed that none of the four IL-23 p19 monoclonal antibodies significantly increased the risk of overall AE or SAE compared with placebo, suggesting that this drug class has a favorable overall safety profile in the induction periods studied. This finding is consistent with previous reviews and long-term studies of IL-23 inhibitors. Previous evidence indicates that IL-23 p19 inhibitors are generally well tolerated in patients with moderate-to-severe plaque psoriasis, with common adverse events including upper respiratory tract infection, nasopharyngitis, headache, and injection-site reactions, while serious adverse events are relatively uncommon (33). However, our quantitative safety synthesis was limited to overall AE and SAE. Specific clinically relevant events, including infections, serious infections, malignancy, injection-site reactions, and gastrointestinal disorders, could not be compared reliably because reporting definitions and event categories were not sufficiently consistent across all included trials.

Previous network meta-analyses have demonstrated that newer biologics, particularly IL-17 and IL-23 pathway inhibitors, are associated with higher skin clearance rates than conventional systemic therapies or some earlier biologics in moderate-to-severe plaque psoriasis (43). A network meta-analysis by Armstrong et al. showed that brodalumab, guselkumab, ixekizumab, and risankizumab were associated with higher PASI response rates, and subsequent NMA studies have also suggested that IL-23-targeted agents may offer a favorable balance between efficacy and safety (44). However, many previous studies evaluated TNF-α inhibitors, IL-17 inhibitors, IL-12/23 inhibitors, and IL-23 inhibitors within a broad biologic treatment network, focusing primarily on differences between biologic classes rather than on within-class differences among IL-23 p19 monoclonal antibodies. In contrast, the present study focused specifically on four IL-23 p19 monoclonal antibodies—guselkumab, risankizumab, tildrakizumab, and mirikizumab—and simultaneously included six categories of outcomes: PASI75, PASI90, PASI100, IGA/PGA/sPGA 0/1, AE, and SAE. Therefore, this study provides a more refined assessment of the relative efficacy and safety profiles within this therapeutic class.

In addition, this study incorporated transitivity assessment, heterogeneity analysis, sensitivity analysis, publication bias assessment, and GRADEpro GDT evidence profiles in the interpretation of results. The transitivity assessment showed that age and sex distributions were generally balanced across treatment groups, supporting the basic assumption for indirect comparisons. Other baseline covariates were considered where available but were incompletely reported across trials. Heterogeneity analysis did not suggest that the overall conclusions were driven by a single study. Leave-one-out sensitivity analyses showed that the directions of the main efficacy effects were stable. Egger’s tests did not indicate obvious publication bias, although power was limited. These methodological assessments improved the interpretability of the network meta-analysis findings and allowed treatment rankings to be interpreted in the context of network structure, baseline comparability, robustness, and certainty of evidence.

### 4.1 Clinical implications

The findings of this study have several implications for clinical practice. First, IL-23 p19 monoclonal antibodies were overall significantly superior to placebo and did not significantly increase the risks of overall AE or SAE, supporting their use as important therapeutic options for achieving deep skin clearance in patients with moderate-to-severe plaque psoriasis. The AAD-NPF biologic treatment guidelines state that biologics have become an important component of systemic therapy for moderate-to-severe psoriasis and emphasize that treatment selection should be individualized according to efficacy, safety, comorbidities, and patient preference (45). Second, risankizumab and guselkumab ranked highly across multiple outcomes, suggesting that they may be considered when treatment goals include high-level efficacy targets such as PASI90, PASI100, or IGA/PGA/sPGA 0/1; however, ranking results should not be used as the sole basis for selecting one agent over another. Third, mirikizumab contributed three RCTs to the plaque psoriasis evidence network and demonstrated favorable efficacy (27–29, 46), but its plaque psoriasis evidence base and regulatory positioning remain less established than those of the other agents.

In actual clinical decision-making, treatment rankings should not be interpreted as the sole basis for drug selection. Psoriasis is a chronic systemic disease requiring long-term management, and treatment decisions should comprehensively consider disease severity, prior treatment failure, comorbidities, dosing frequency, patient adherence, economic burden, and drug accessibility. In the long-term maintenance phase, safety, dosing convenience, and improvement in quality of life are also important considerations. Therefore, the findings of this study should be viewed as evidence-based support for individualized biologic selection rather than as a substitute for clinical judgment.

## 5 Limitations

This study has several limitations. First, the number of included studies was limited. Although 15 RCTs or independent study units were included, the amount of evidence was not evenly distributed across drugs and outcomes, and some outcomes had wide CrIs, indicating imprecision in the estimates. Second, the network structure was mainly centered on placebo, with insufficient direct head-to-head comparisons among active agents; therefore, the relative efficacy comparisons among different IL-23 p19 monoclonal antibodies were mainly based on indirect evidence and should be interpreted as exploratory. Third, the included studies varied in follow-up duration, dosing regimen, study region, and baseline characteristics, and some baseline covariates were incompletely reported. Fourth, the safety synthesis was limited to overall AE and SAE and did not allow robust comparison of specific adverse events such as infections, serious infections, malignancy, injection-site reactions, or gastrointestinal disorders. Fifth, this study was primarily based on RCT data; therefore, long-term efficacy, safety, relapse after treatment withdrawal, switching strategies, and cost-effectiveness in more complex real-world patient populations were not fully evaluated.

## 6 Conclusion

In summary, this study indicates that IL-23 p19 monoclonal antibodies provide clear clinical benefit in moderate-to-severe plaque psoriasis, improving PASI75, PASI90, PASI100, and IGA/PGA/sPGA 0/1 response rates without a clear increase in overall AE or SAE risk compared with placebo. Treatment ranking results suggest that risankizumab has the highest probability of favorable efficacy across multiple outcomes, followed by guselkumab and mirikizumab, while tildrakizumab ranks relatively lower but remains superior to placebo for several outcomes. Because active-agent comparisons are mainly indirect and uncertainty remains for sparse outcomes and specific safety events, these rankings should be interpreted as exploratory rather than definitive. Future head-to-head RCTs, specific adverse-event analyses, and long-term real-world studies are needed to clarify differences among IL-23 p19 monoclonal antibodies in population subgroups, long-term safety, treatment persistence, and cost-effectiveness.

## Data Availability

The original contributions presented in the study are included in the article/**Supplementary Material**. Further inquiries can be directed to the corresponding author.
