# Supplementary material for: Comparative efficacy and safety of IL-23 p19 monoclonal antibodies for plaque psoriasis: a bayesian network meta-analysis of randomized controlled trials
Source: Front Immunol. 2026 Aug 12;17:1873488. doi: 10.3389/fimmu.2026.1873488 (PMC13506889; doi:10.3389/fimmu.2026.1873488)
Supplement: Supplementary file 1 [file Table1.docx]

**Detailed Search Strategies for Each Database**

**1.Cochrane Library**

Search Terms

#1 MeSH descriptor: [Psoriasis] explode all trees

#2 (psoria* OR "plaque psoriasis" OR "psoriasis vulgaris" OR "chronic plaque psoriasis"):ti,ab,kw

#3 #1 OR #2

#4 (guselkumab OR risankizumab OR tildrakizumab OR mirikizumab OR picankibart OR BI655066 OR "BI 655066" OR CNTO1959 OR "CNTO 1959" OR MK3222 OR "MK-3222" OR LY3074828 OR IBI112 OR Tremfya OR Skyrizi OR Ilumya OR Ilumetri OR Omvoh OR Pecondle):ti,ab,kw

#5 (("interleukin 23" near/3 p19) OR "IL-23 p19" OR IL23p19 OR "anti-IL-23 p19"):ti,ab,kw

#6 #4 OR #5

#7 (random* OR placebo* OR trial OR "double blind" OR double-blind OR multicenter OR multicentre):ti,ab,kw

#8 #3 AND #6 AND #7

**2.Web of Science**

Search Terms

TS=(

(psoria* OR "plaque psoriasis" OR "psoriasis vulgaris" OR "chronic plaque psoriasis")

AND

(guselkumab OR risankizumab OR tildrakizumab OR mirikizumab OR picankibart

OR BI655066 OR "BI 655066"

OR CNTO1959 OR "CNTO 1959"

OR MK3222 OR "MK-3222"

OR LY3074828 OR IBI112

OR Tremfya OR Skyrizi OR Ilumya OR Ilumetri OR Omvoh OR Pecondle

OR ("interleukin 23" NEAR/3 p19)

OR "IL-23 p19" OR IL23p19 OR "anti-IL-23 p19")

AND

(random* OR placebo* OR trial OR "double blind*" OR "double-blind*" OR multicenter OR multicentre)

)

**3. Embase**

Search Terms

(

'psoriasis'/exp OR psoria*:ti,ab,kw OR 'plaque psoriasis':ti,ab,kw OR 'psoriasis vulgaris':ti,ab,kw OR 'chronic plaque psoriasis':ti,ab,kw

)

AND

(

guselkumab:ti,ab,kw OR risankizumab:ti,ab,kw OR tildrakizumab:ti,ab,kw OR mirikizumab:ti,ab,kw OR picankibart:ti,ab,kw OR bi655066:ti,ab,kw OR 'bi 655066':ti,ab,kwOR cnto1959:ti,ab,kw OR 'cnto 1959':ti,ab,kw OR mk3222:ti,ab,kw OR 'mk-3222':ti,ab,kwOR ly3074828:ti,ab,kw OR ibi112:ti,ab,kw OR tremfya:ti,ab,kw OR skyrizi:ti,ab,kw OR ilumya:ti,ab,kw OR ilumetri:ti,ab,kw OR omvoh:ti,ab,kw OR pecondle:ti,ab,kw OR (('interleukin 23'/exp OR 'interleukin 23':ti,ab,kw) AND p19:ti,ab,kw) OR 'il 23 p19':ti,ab,kw OR il23p19:ti,ab,kw OR 'anti il 23 p19':ti,ab,kw

)

AND

(

'randomized controlled trial'/de OR 'clinical trial'/de OR 'double blind procedure'/de OR random*:ti,ab,kw OR placebo*:ti,ab,kw OR trial:ti,ab,kw OR 'double blind*':ti,ab,kw OR 'double-blind*':ti,ab,kw

)

**4. Pubmed**

Search Terms

(

"Psoriasis"[Mesh]

OR psoria*[tiab]

OR "plaque psoriasis"[tiab]

OR "psoriasis vulgaris"[tiab]

OR "chronic plaque psoriasis"[tiab]

)

AND

(

guselkumab[tiab]

OR risankizumab[tiab]

OR tildrakizumab[tiab]

OR mirikizumab[tiab]

OR picankibart[tiab]

OR BI655066[tiab]

OR CNTO1959[tiab]

OR MK3222[tiab]

OR LY3074828[tiab]

OR IBI112[tiab]

OR Tremfya[tiab]

OR Skyrizi[tiab]

OR Ilumya[tiab]

OR Ilumetri[tiab]

OR Omvoh[tiab]

OR Pecondle[tiab]

OR (("interleukin 23"[tiab] OR "IL-23"[tiab]) AND p19[tiab])

OR "IL-23 p19"[tiab]

OR IL23p19[tiab]

OR "anti-IL-23 p19"[tiab]

)

AND

(

"Randomized Controlled Trial"[Publication Type]

OR "Clinical Trial"[Publication Type]

OR random*[tiab]

OR placebo*[tiab]

OR "double blind"[tiab]

OR "double-blind"[tiab]

)

NOT

(

animals[mh] NOT humans[mh]

)

**Supplementary Results Added for Revision**

The following tables were added in response to reviewer comments. They summarize the existing network meta-analysis league tables, SUCRA values, and outcome extraction time points. Detailed GRADEpro GDT evidence profiles for the 10 key comparisons are provided separately in Supplementary_GRADE_evidence_profiles.docx. These materials are derived from the existing analysis outputs and do not change the original effect estimates.

**Supplementary Table S1. League tables for network meta-analysis outcomes**

**S1.PASI90. PASI90**

| **Treatment** | **Placebo** | **Guselkumab** | **Risankizumab** | **Tildrakizumab** | **Mirikizumab** |
| --- | --- | --- | --- | --- | --- |
| **Placebo** | **Placebo** | 117.47 (8.41, 2093.11) | 145.83 (12.07, 1866.23) | 6.34 (0.55, 78.88) | 91.79 (5.59, 2376.62) |
| Guselkumab | 0.01 (0, 0.12) | Guselkumab | 1.24 (0.03, 46.86) | 0.05 (0, 2.05) | 0.78 (0.01, 50.52) |
| Risankizumab | 0.01 (0, 0.08) | 0.81 (0.02, 36.4) | Risankizumab | 0.04 (0, 1.49) | 0.63 (0.01, 39.37) |
| Tildrakizumab | 0.16 (0.01, 1.81) | 18.5 (0.49, 811.67) | 22.83 (0.67, 782.3) | Tildrakizumab | 14.48 (0.34, 877.51) |
| Mirikizumab | 0.01 (0, 0.18) | 1.28 (0.02, 67.88) | 1.58 (0.03, 68.33) | 0.07 (0, 2.9) | Mirikizumab |

**S1.PASI75. PASI75**

| **Treatment** | **Placebo** | **Guselkumab** | **Risankizumab** | **Tildrakizumab** | **Mirikizumab** |
| --- | --- | --- | --- | --- | --- |
| **Placebo** | **Placebo** | 104.09 (53.36, 219.34) | 121.85 (48.4, 347.64) | 23.82 (13.45, 45.67) | 71.89 (35.7, 153.19) |
| Guselkumab | 0.01 (0, 0.02) | Guselkumab | 1.17 (0.36, 4.06) | 0.23 (0.09, 0.58) | 0.69 (0.25, 1.88) |
| Risankizumab | 0.01 (0, 0.02) | 0.86 (0.25, 2.8) | Risankizumab | 0.2 (0.06, 0.6) | 0.59 (0.17, 1.9) |
| Tildrakizumab | 0.04 (0.02, 0.07) | 4.37 (1.72, 11.13) | 5.1 (1.66, 16.7) | Tildrakizumab | 3.02 (1.16, 7.69) |
| Mirikizumab | 0.01 (0.01, 0.03) | 1.45 (0.53, 4.05) | 1.69 (0.53, 6.02) | 0.33 (0.13, 0.86) | Mirikizumab |

**S1.PASI100. PASI100**

| **Treatment** | **Placebo** | **Guselkumab** | **Risankizumab** | **Tildrakizumab** | **Mirikizumab** |
| --- | --- | --- | --- | --- | --- |
| **Placebo** | **Placebo** | 109.63 (24.49, 1321.07) | 126.56 (32.6, 1548.07) | 42.8 (8.83, 1605.44) | 63.98 (14.1, 805.73) |
| Guselkumab | 0.01 (0, 0.04) | Guselkumab | 1.16 (0.09, 18.48) | 0.39 (0.03, 15.06) | 0.58 (0.04, 9.41) |
| Risankizumab | 0.01 (0, 0.03) | 0.86 (0.05, 11.71) | Risankizumab | 0.35 (0.02, 10.34) | 0.5 (0.03, 7.06) |
| Tildrakizumab | 0.02 (0, 0.11) | 2.54 (0.07, 34.14) | 2.83 (0.1, 41.72) | Tildrakizumab | 1.45 (0.04, 21.36) |
| Mirikizumab | 0.02 (0, 0.07) | 1.72 (0.11, 26.59) | 2.01 (0.14, 33.47) | 0.69 (0.05, 22.69) | Mirikizumab |

**S1.PGA. IGA/PGA/sPGA 0/1**

| **Treatment** | **Placebo** | **Guselkumab** | **Risankizumab** | **Tildrakizumab** | **Mirikizumab** |
| --- | --- | --- | --- | --- | --- |
| **Placebo** | **Placebo** | 68.29 (42.65, 116.95) | 89.29 (53.83, 156.32) | 21.23 (13.55, 36.13) | 52.14 (28.84, 102.7) |
| Guselkumab | 0.01 (0.01, 0.02) | Guselkumab | 1.31 (0.63, 2.74) | 0.31 (0.16, 0.62) | 0.76 (0.35, 1.75) |
| Risankizumab | 0.01 (0.01, 0.02) | 0.76 (0.37, 1.59) | Risankizumab | 0.24 (0.12, 0.5) | 0.59 (0.26, 1.38) |
| Tildrakizumab | 0.05 (0.03, 0.07) | 3.2 (1.6, 6.38) | 4.2 (2.02, 8.57) | Tildrakizumab | 2.45 (1.11, 5.47) |
| Mirikizumab | 0.02 (0.01, 0.03) | 1.32 (0.57, 2.89) | 1.71 (0.73, 3.87) | 0.41 (0.18, 0.9) | Mirikizumab |

**S1.AE. Any adverse event**

| **Treatment** | **Placebo** | **Guselkumab** | **Risankizumab** | **Tildrakizumab** | **Mirikizumab** |
| --- | --- | --- | --- | --- | --- |
| **Placebo** | **Placebo** | 1.08 (0.82, 1.41) | 0.94 (0.71, 1.24) | 0.84 (0.64, 1.11) | 0.94 (0.69, 1.29) |
| Guselkumab | 0.93 (0.71, 1.22) | Guselkumab | 0.87 (0.59, 1.29) | 0.78 (0.53, 1.15) | 0.87 (0.58, 1.33) |
| Risankizumab | 1.07 (0.81, 1.41) | 1.15 (0.78, 1.69) | Risankizumab | 0.89 (0.61, 1.33) | 1 (0.65, 1.54) |
| Tildrakizumab | 1.19 (0.9, 1.56) | 1.28 (0.87, 1.87) | 1.12 (0.75, 1.64) | Tildrakizumab | 1.12 (0.73, 1.69) |
| Mirikizumab | 1.06 (0.78, 1.46) | 1.15 (0.75, 1.72) | 1 (0.65, 1.53) | 0.89 (0.59, 1.36) | Mirikizumab |

**S1.SAE. Serious adverse event**

| **Treatment** | **Placebo** | **Guselkumab** | **Risankizumab** | **Tildrakizumab** | **Mirikizumab** |
| --- | --- | --- | --- | --- | --- |
| **Placebo** | **Placebo** | 1.26 (0.36, 4.8) | 0.67 (0.25, 2.3) | 0.95 (0.27, 3.84) | 1.7 (0.38, 10.82) |
| Guselkumab | 0.79 (0.21, 2.81) | Guselkumab | 0.53 (0.1, 3.24) | 0.75 (0.12, 5.08) | 1.36 (0.18, 12.58) |
| Risankizumab | 1.49 (0.43, 3.98) | 1.87 (0.31, 9.6) | Risankizumab | 1.4 (0.24, 7.55) | 2.52 (0.36, 20.08) |
| Tildrakizumab | 1.05 (0.26, 3.7) | 1.34 (0.2, 8.21) | 0.71 (0.13, 4.1) | Tildrakizumab | 1.78 (0.23, 16.56) |
| Mirikizumab | 0.59 (0.09, 2.65) | 0.74 (0.08, 5.49) | 0.4 (0.05, 2.76) | 0.56 (0.06, 4.31) | Mirikizumab |

Supplementary Table S2. Selected extraction time points and comparator arms

| **Study/trial unit** | **Intervention arm used** | **Comparator arm used** | **Selected extraction time point** | **Reported main outcomes/time points** |
| --- | --- | --- | --- | --- |
| Kenneth B. Gordon—2015[19] | Guselkumab 100 mg | Placebo | Week 16 for efficacy outcomes in the placebo-controlled induction period | Week 16 PGA 0/1, PASI75, PASI90, PASI100; AE, SAE |
| Andrew Blauvelt—2017, VOYAGE 1[9] | Guselkumab 100 mg | Placebo | Week 16 for efficacy outcomes in the placebo-controlled induction period | Week 16 IGA 0/1, PASI90, PASI75, PASI100; AE, SAE |
| Kristian Reich—2017, VOYAGE 2[20] | Guselkumab 100 mg | Placebo | Week 16 for efficacy outcomes in the placebo-controlled induction period | Week 16 IGA 0/1, PASI90, PASI75, PASI100; AE, SAE |
| O. Nemoto—2018[21] | Guselkumab 100 mg | Placebo | Week 16 for efficacy outcomes in the placebo-controlled induction period | Week 16 PASI, PGA, safety, pharmacokinetics, immunogenicity |
| Kenneth B. Gordon—2018, UltIMMa-1[8] | Risankizumab 150 mg | Placebo | Week 16 for efficacy outcomes in the placebo-controlled induction period | Week 16 PASI90, sPGA 0/1, PASI75, PASI100; AE, SAE |
| Kenneth B. Gordon—2018, UltIMMa-2[8] | Risankizumab 150 mg | Placebo | Week 16 for efficacy outcomes in the placebo-controlled induction period | Week 16 PASI90, sPGA 0/1, PASI75, PASI100; AE, SAE |
| Mamitaro Ohtsuki—2019, SustaIMM[22] | Risankizumab 150 mg | Placebo | Week 16 for efficacy outcomes in the placebo-controlled induction period | Week 16 PASI90, PASI75, PASI100, sPGA 0/1; AE, SAE |
| Andrew Blauvelt—2020[23] | Risankizumab 150 mg | Placebo | Week 16 for efficacy outcomes in the placebo-controlled induction period | Week 16 PASI90, sPGA 0/1, PASI75, PASI100; long-term maintenance outcomes; AE, SAE |
| K. Papp—2015[24] | Tildrakizumab 100 mg | Placebo | Week 12 for efficacy outcomes in the placebo-controlled induction period | Week 12 PASI75, PASI90, PGA clear/minimal, DLQI; AE, SAE |
| Kristian Reich—2017, reSURFACE 1[25] | Tildrakizumab 100 mg | Placebo | Week 12 for efficacy outcomes in the placebo-controlled induction period | Week 12 PASI75, PGA 0/1, PASI90, PASI100; AE, SAE |
| Kristian Reich—2017, reSURFACE 2[25] | Tildrakizumab 100 mg | Placebo | Week 12 for efficacy outcomes in the placebo-controlled induction period | Week 12 PASI75, PGA response, PASI90, PASI100; AE, SAE |
| Chen Yu—2024[26] | Tildrakizumab 100 mg | Placebo | Week 12 for efficacy outcomes in the placebo-controlled induction period | Week 12 PASI75, PGA response, PASI90, PASI100; AE, SAE |
| Kim Papp—2023, OASIS-2[27] | Mirikizumab 250 mg | Placebo | Week 16 for efficacy outcomes in the placebo-controlled induction period | Week 16 sPGA 0/1, PASI90, PASI75, PASI100; week 52 long-term outcomes; AE, SAE |
| Andrew Blauvelt—2022, OASIS-1[28] | Mirikizumab 250 mg | Placebo | Week 16 for efficacy outcomes in the placebo-controlled induction period | Week 16 sPGA 0/1, PASI90, PASI75, PASI100; maintenance outcomes; AE, SAE |
| Kristian Reich—2019[29] | Mirikizumab 300 mg | Placebo | Week 16 for efficacy outcomes in the placebo-controlled induction period | Week 16 PASI90, PASI75, PASI100, sPGA 0/1, BSA≤1%, PSSI, PSS, DLQI; AE, SAE |
