# Supplementary material for: Comparative efficacy and safety of IL-23 p19 monoclonal antibodies for plaque psoriasis: a bayesian network meta-analysis of randomized controlled trials
Source: Front Immunol. 2026 Aug 12;17:1873488. doi: 10.3389/fimmu.2026.1873488 (PMC13506889; doi:10.3389/fimmu.2026.1873488)
Supplement: Supplementary file 2 [file Table2.docx]

# Supplementary GRADE Evidence Profiles

IL-23 p19 monoclonal antibodies for plaque psoriasis

## Profile 1. Guselkumab compared to Placebo for plaque psoriasis

| **Outcome** | **No. of studies** | **Study design** | **Risk of bias** | **Inconsistency** | **Indirectness** | **Imprecision** | **Other considerations** | **Certainty** |
| --- | --- | --- | --- | --- | --- | --- | --- | --- |
| PASI90 | 0 | randomised trials | not serious | not serious | not serious | serious | none | Moderate |
| PASI75 | 0 | randomised trials | not serious | not serious | not serious | not serious | none | High |
| PASI100 | 0 | randomised trials | not serious | not serious | not serious | serious | none | Moderate |
| IGA/PGA/sPGA | 0 | randomised trials | not serious | not serious | not serious | not serious | none | High |
| AE | 0 | randomised trials | not serious | not serious | not serious | serious | none | Moderate |
| SAE | 0 | randomised trials | not serious | not serious | not serious | serious | none | Moderate |

| **Outcome** | **Guselkumab** | **Placebo** | **Relative effect (95% CrI)** | **Absolute effect (95% CrI)** | **Certainty** |
| --- | --- | --- | --- | --- | --- |
| PASI90 | 616/872 (70.6%) | 81/1558 (5.2%) | OR 117.47 (8.41 to 2093.11) | 813,641 more per 1,000,000 (from 263,647 more to 939,374 more) | Moderate |
| PASI75 | 764/872 (87.6%) | 100/1358 (7.4%) | OR 104.09 (53.36 to 219.34) | 819 more per 1,000 (from 736 more to 872 more) | High |
| PASI100 | 306/867 (35.3%) | 11/1513 (0.7%) | OR 109.63 (24.49 to 1321.07) | 438 more per 1,000 (from 145 more to 899 more) | Moderate |
| IGA/PGA/sPGA | 737/872 (84.5%) | 107/1555 (6.9%) | OR 68.29 (42.65 to 116.95) | 766 more per 1,000 (from 690 more to 827 more) | High |
| AE | 511/1037 (49.3%) | 801/1558 (51.4%) | OR 1.08 (0.82 to 1.41) | 19 more per 1,000 (from 50 fewer to 85 more) | Moderate |
| SAE | 19/1037 (1.8%) | 30/1558 (1.9%) | OR 1.26 (0.36 to 4.80) | 5 more per 1,000 (from 12 fewer to 67 more) | Moderate |

## Profile 2. Risankizumab compared to Placebo for plaque psoriasis

| **Outcome** | **No. of studies** | **Study design** | **Risk of bias** | **Inconsistency** | **Indirectness** | **Imprecision** | **Other considerations** | **Certainty** |
| --- | --- | --- | --- | --- | --- | --- | --- | --- |
| PASI90 | 0 | randomised trials | not serious | not serious | not serious | serious | none | Moderate |
| PASI75 | 0 | randomised trials | not serious | not serious | not serious | not serious | none | High |
| PASI100 | 0 | randomised trials | not serious | not serious | not serious | serious | none | Moderate |
| IGA/PGA/sPGA | 0 | randomised trials | not serious | not serious | not serious | not serious | none | High |
| AE | 0 | randomised trials | not serious | not serious | not serious | serious | none | Moderate |
| SAE | 0 | randomised trials | not serious | not serious | not serious | serious | none | Moderate |

| **Outcome** | **Risankizumab** | Placebo | **Relative effect (95% CrI)** | **Absolute effect (95% CrI)** | **Certainty** |
| --- | --- | --- | --- | --- | --- |
| PASI90 | 788/1060 (74.3%) | 81/1558 (5.2%) | OR 145.83 (12.07 to 1866.23) | 837 more per 1,000 (from 346 more to 938 more) | Moderate |
| PASI75 | 413/462 (89.4%) | 100/1358 (7.4%) | OR 121.85 (48.40 to 347.64) | 833 more per 1,000 (from 720 more to 891 more) | High |
| PASI100 | 468/1060 (44.2%) | 11/1513 (0.7%) | OR 126.56 (32.60 to 1548.07) | 474 more per 1,000 (from 185 more to 912 more) | Moderate |
| IGA/PGA/sPGA | 904/1060 (85.3%) | 107/1555 (6.9%) | OR 89.29 (53.83 to 156.32) | 800 more per 1,000 (from 730 more to 852 more) | High |
| AE | 502/1060 (47.4%) | 801/1558 (51.4%) | OR 0.94 (0.71 to 1.24) | 15 fewer per 1,000 (from 85 fewer to 53 more) | Moderate |
| SAE | 23/1060 (2.2%) | 30/1558 (1.9%) | OR 0.67 (0.25 to 2.30) | 6 fewer per 1,000 (from 14 fewer to 24 more) | Moderate |

## Profile 3. Tildrakizumab compared to Placebo for plaque psoriasis

| **Outcome** | **No. of studies** | **Study design** | **Risk of bias** | **Inconsistency** | **Indirectness** | **Imprecision** | **Other considerations** | **Certainty** |
| --- | --- | --- | --- | --- | --- | --- | --- | --- |
| PASI90 | 0 | randomised trials | not serious | not serious | not serious | serious | none | Moderate |
| PASI75 | 0 | randomised trials | not serious | not serious | not serious | not serious | none | High |
| PASI100 | 0 | randomised trials | not serious | not serious | not serious | serious | none | Moderate |
| IGA/PGA/sPGA | 0 | randomised trials | not serious | not serious | not serious | not serious | none | High |
| AE | 0 | randomised trials | not serious | not serious | not serious | serious | none | Moderate |
| SAE | 0 | randomised trials | not serious | not serious | not serious | serious | none | Moderate |

| **Outcome** | **Tildrakizumab** | **Placebo** | **Relative effect (95% CrI)** | **Absolute effect (95% CrI)** | **Certainty** |
| --- | --- | --- | --- | --- | --- |
| PASI90 | 263/815 (32.3%) | 81/1558 (5.2%) | OR 6.34 (0.55 to 78.88) | 206 more per 1,000 (from 23 fewer to 760 more) | Moderate |
| PASI75 | 517/815 (63.4%) | 1358/100 (1358.0%) | OR 23.82 (13.45 to 45.67) | 1,000 fewer per 1,000 (from 1,000 fewer to 1,000 fewer) | High |
| PASI100 | 89/726 (12.3%) | 11/1513 (0.7%) | OR 42.80 (8.83 to 1605.44) | 231 more per 1,000 (from 53 more to 914 more) | Moderate |
| IGA/PGA/sPGA | 469/815 (57.5%) | 107/1555 (6.9%) | OR 21.23 (13.55 to 36.13) | 542 more per 1,000 (from 432 more to 659 more) | High |
| AE | 411/815 (50.4%) | 801/1558 (51.4%) | OR 0.84 (0.64 to 1.11) | 44 fewer per 1,000 (from 110 fewer to 26 more) | Moderate |
| SAE | 11/815 (1.3%) | 30/1558 (1.9%) | OR 0.95 (0.27 to 3.84) | 1 fewer per 1,000 (from 14 fewer to 51 more) | Moderate |

## Profile 4. Mirikizumab compared to Placebo for plaque psoriasis

| **Outcome** | **No. of studies** | **Study design** | **Risk of bias** | **Inconsistency** | **Indirectness** | **Imprecision** | **Other considerations** | **Certainty** |
| --- | --- | --- | --- | --- | --- | --- | --- | --- |
| PASI90 | 0 | randomised trials | not serious | not serious | not serious | serious | none | Moderate |
| PASI75 | 0 | randomised trials | not serious | not serious | not serious | not serious | none | High |
| PASI100 | 0 | randomised trials | not serious | not serious | not serious | serious | none | Moderate |
| IGA/PGA/sPGA | 0 | randomised trials | not serious | not serious | not serious | not serious | none | High |
| AE | 0 | randomised trials | not serious | not serious | not serious | serious | none | Moderate |
| SAE | 0 | randomised trials | not serious | not serious | not serious | serious | none | Moderate |

| **Outcome** | **Mirikizumab** | **Placebo** | **Relative effect (95% CrI)** | **Absolute effect (95% CrI)** | **Certainty** |
| --- | --- | --- | --- | --- | --- |
| PASI90 | 979/1379 (71.0%) | 81/1558 (5.2%) | OR 91.79 (5.59 to 2376.62) | 782 more per 1,000 (from 183 more to 940 more) | Moderate |
| PASI75 | 1197/1379 (86.8%) | 100/1358 (7.4%) | OR 71.89 (35.70 to 153.19) | 777 more per 1,000 (from 666 more to 850 more) | High |
| PASI100 | 494/1379 (35.8%) | 11/1513 (0.7%) | OR 63.98 (14.10 to 805.73) | 312 more per 1,000 (from 86 more to 848 more) | Moderate |
| IGA/PGA/sPGA | 1049/1379 (76.1%) | 107/1555 (6.9%) | OR 52.14 (28.84 to 102.70) | 725 more per 1,000 (from 612 more to 815 more) | High |
| AE | 749/1379 (54.3%) | 801/1558 (51.4%) | OR 0.94 (0.69 to 1.29) | 15 fewer per 1,000 (from 92 fewer to 63 more) | Moderate |
| SAE | 21/1379 (1.5%) | 30/1558 (1.9%) | OR 1.70 (0.38 to 10.82) | 13 more per 1,000 (from 12 fewer to 156 more) | Moderate |

## Profile 5. Risankizumab compared to Guselkumab for plaque psoriasis

| **Outcome** | **No. of studies** | **Study design** | **Risk of bias** | **Inconsistency** | **Indirectness** | **Imprecision** | **Other considerations** | **Certainty** |
| --- | --- | --- | --- | --- | --- | --- | --- | --- |
| PASI90 | 0 | randomised trials | not serious | not serious | serious | not serious | none | Moderate |
| PASI75 | 0 | randomised trials | not serious | not serious | serious | not serious | none | Moderate |
| PASI100 | 0 | randomised trials | not serious | not serious | serious | not serious | none | Moderate |
| IGA/PGA/sPGA | 0 | randomised trials | not serious | not serious | serious | not serious | none | Moderate |
| AE | 0 | randomised trials | not serious | not serious | serious | not serious | none | Moderate |
| SAE | 0 | randomised trials | not serious | not serious | serious | not serious | none | Moderate |

| **Outcome** | **Risankizumab** | **Guselkumab** | **Relative effect (95% CrI)** | **Absolute effect (95% CrI)** | **Certainty** |
| --- | --- | --- | --- | --- | --- |
| PASI90 | 788/1060 (74.3%) | 616/872 (70.6%) | OR 1.24 (0.03 to 46.86) | 43 more per 1,000 (from 639 fewer to 285 more) | Moderate |
| PASI75 | 413/462 (89.4%) | 764/872 (87.6%) | OR 1.17 (0.36 to 4.06) | 16 more per 1,000 (from 158 fewer to 90 more) | Moderate |
| PASI100 | 468/1060 (44.2%) | 306/867 (35.3%) | OR 1.16 (0.09 to 18.48) | 35 more per 1,000 (from 306 fewer to 557 more) | Moderate |
| IGA/PGA/sPGA | 904/1060 (85.3%) | 737/872 (84.5%) | OR 1.31 (0.63 to 2.74) | 32 more per 1,000 (from 70 fewer to 92 more) | Moderate |
| AE | 502/1060 (47.4%) | 511/1037 (49.3%) | OR 0.87 (0.59 to 1.29) | 35 fewer per 1,000 (from 128 fewer to 63 more) | Moderate |
| SAE | 23/1060 (2.2%) | 19/1037 (1.8%) | OR 0.53 (0.10 to 3.24) | 9 fewer per 1,000 (from 16 fewer to 39 more) | Moderate |

## Profile 6. Tildrakizumab compared to Guselkumab for plaque psoriasis

| **Outcome** | **No. of studies** | **Study design** | **Risk of bias** | **Inconsistency** | **Indirectness** | **Imprecision** | **Other considerations** | **Certainty** |
| --- | --- | --- | --- | --- | --- | --- | --- | --- |
| PASI90 | 0 | randomised trials | not serious | not serious | serious | not serious | none | Moderate |
| PASI75 | 0 | randomised trials | not serious | not serious | serious | not serious | none | Moderate |
| PASI100 | 0 | randomised trials | not serious | not serious | serious | not serious | none | Moderate |
| PGA. IGA/PGA/sPGA | 0 | randomised trials | not serious | not serious | serious | not serious | none | Moderate |
| AE | 0 | randomised trials | not serious | not serious | serious | not serious | none | Moderate |
| SAE | 0 | randomised trials | not serious | not serious | serious | not serious | none | Moderate |

| **Outcome** | **Tildrakizumab** | **Guselkumab** | **Relative effect (95% CrI)** | **Absolute effect (95% CrI)** | **Certainty** |
| --- | --- | --- | --- | --- | --- |
| PASI90 | 263/815 (32.3%) | 616/872 (70.6%) | OR 0.05 (0.00 to 2.05) | 599 fewer per 1,000 (from -- to 125 more) | Moderate |
| PASI75 | 517/815 (63.4%) | 764/872 (87.6%) | OR 0.23 (0.09 to 0.58) | 257 fewer per 1,000 (from 487 fewer to 72 fewer) | Moderate |
| PASI100 | 89/726 (12.3%) | 306/867 (35.3%) | OR 0.39 (0.03 to 15.06) | 178 fewer per 1,000 (from 337 fewer to 539 more) | Moderate |
| PGA. IGA/PGA/sPGA | 469/815 (57.5%) | 737/872 (84.5%) | OR 0.31 (0.16 to 0.62) | 217 fewer per 1,000 (from 379 fewer to 73 fewer) | Moderate |
| AE | 411/815 (50.4%) | 511/1037 (49.3%) | OR 0.78 (0.53 to 1.15) | 62 fewer per 1,000 (from 153 fewer to 35 more) | Moderate |
| SAE | 11/815 (1.3%) | 19/1037 (1.8%) | OR 0.75 (0.12 to 5.08) | 5 fewer per 1,000 (from 16 fewer to 68 more) | Moderate |

## Profile 7. Mirikizumab compared to Guselkumab for plaque psoriasis

| **Outcome** | **No. of studies** | **Study design** | **Risk of bias** | **Inconsistency** | **Indirectness** | **Imprecision** | **Other considerations** | **Certainty** |
| --- | --- | --- | --- | --- | --- | --- | --- | --- |
| PASI90 | 0 | randomised trials | not serious | not serious | serious | not serious | none | Moderate |
| PASI75 | 0 | randomised trials | not serious | not serious | serious | not serious | none | Moderate |
| PASI100 | 0 | randomised trials | not serious | not serious | serious | not serious | none | Moderate |
| PGA/IGA/PGA/sPGA | 0 | randomised trials | not serious | not serious | serious | not serious | none | Moderate |
| AE | 0 | randomised trials | not serious | not serious | serious | not serious | none | Moderate |
| SAE | 0 | randomised trials | not serious | not serious | serious | not serious | none | Moderate |

| **Outcome** | **Mirikizumab** | **Guselkumab** | **Relative effect (95% CrI)** | **Absolute effect (95% CrI)** | **Certainty** |
| --- | --- | --- | --- | --- | --- |
| PASI90 | 979/1379 (71.0%) | 616/872 (70.6%) | OR 0.78 (0.01 to 50.52) | 54 fewer per 1,000 (from 683 fewer to 285 more) | Moderate |
| PASI75 | 1197/1379 (86.8%) | 764/872 (87.6%) | OR 0.69 (0.25 to 1.88) | 46 fewer per 1,000 (from 237 fewer to 54 more) | Moderate |
| PASI100 | 494/1379 (35.8%) | 306/867 (35.3%) | OR 0.58 (0.04 to 9.41) | 113 fewer per 1,000 (from 332 fewer to 484 more) | Moderate |
| PGA/IGA/PGA/sPGA | 1049/1379 (76.1%) | 737/872 (84.5%) | OR 0.76 (0.35 to 1.75) | 39 fewer per 1,000 (from 189 fewer to 60 more) | Moderate |
| AE | 749/1379 (54.3%) | 511/1037 (49.3%) | OR 0.87 (0.58 to 1.33) | 35 fewer per 1,000 (from 132 fewer to 71 more) | Moderate |
| SAE | 21/1379 (1.5%) | 19/1037 (1.8%) | OR 1.36 (0.18 to 12.58) | 6 more per 1,000 (from 15 fewer to 172 more) | Moderate |

## Profile 8. Tildrakizumab compared to Risankizumab for plaque psoriasis

| **Outcome** | **No. of studies** | **Study design** | **Risk of bias** | **Inconsistency** | **Indirectness** | **Imprecision** | **Other considerations** | **Certainty** |
| --- | --- | --- | --- | --- | --- | --- | --- | --- |
| PASI90 | 0 | randomised trials | not serious | not serious | serious | not serious | none | Moderate |
| PASI75 | 0 | randomised trials | not serious | not serious | serious | not serious | none | Moderate |
| PASI100 | 0 | randomised trials | not serious | not serious | serious | not serious | none | Moderate |
| IGA/PGA/sPGA | 0 | randomised trials | not serious | not serious | serious | not serious | none | Moderate |
| AE | 0 | randomised trials | not serious | not serious | serious | not serious | none | Moderate |
| SAE | 0 | randomised trials | not serious | not serious | serious | not serious | none | Moderate |

| **Outcome** | **Tildrakizumab** | **Risankizumab** | **Relative effect (95% CrI)** | **Absolute effect (95% CrI)** | **Certainty** |
| --- | --- | --- | --- | --- | --- |
| PASI90 | 263/815 (32.3%) | 788/1060 (74.3%) | OR 0.04 (0.00 to 1.49) | 640 fewer per 1,000 (from -- to 69 more) | Moderate |
| PASI75 | 517/815 (63.4%) | 413/462 (89.4%) | OR 0.20 (0.06 to 0.60) | 266 fewer per 1,000 (from 558 fewer to 59 fewer) | Moderate |
| PASI100 | 89/726 (12.3%) | 468/1060 (44.2%) | OR 0.35 (0.02 to 10.34) | 225 fewer per 1,000 (from 426 fewer to 449 more) | Moderate |
| IGA/PGA/sPGA | 469/815 (57.5%) | 904/1060 (85.3%) | OR 0.24 (0.12 to 0.50) | 271 fewer per 1,000 (from 443 fewer to 109 fewer) | Moderate |
| AE | 411/815 (50.4%) | 502/1060 (47.4%) | OR 0.89 (0.61 to 1.33) | 29 fewer per 1,000 (from 119 fewer to 71 more) | Moderate |
| SAE | 11/815 (1.3%) | 23/1060 (2.2%) | OR 1.40 (0.24 to 7.55) | 8 more per 1,000 (from 16 fewer to 122 more) | Moderate |

## Profile 9. Mirikizumab compared to Risankizumab for plaque psoriasis

| **Outcome** | **No. of studies** | **Study design** | **Risk of bias** | **Inconsistency** | **Indirectness** | **Imprecision** | **Other considerations** | **Certainty** |
| --- | --- | --- | --- | --- | --- | --- | --- | --- |
| PASI90 | 0 | randomised trials | not serious | not serious | serious | not serious | none | Moderate |
| PASI75 | 0 | randomised trials | not serious | not serious | serious | not serious | none | Moderate |
| PASI100 | 0 | randomised trials | not serious | not serious | serious | not serious | none | Moderate |
| IGA/PGA/sPGA | 0 | randomised trials | not serious | not serious | serious | not serious | none | Moderate |
| AE | 0 | randomised trials | not serious | not serious | serious | not serious | none | Moderate |
| SAE | 0 | randomised trials | not serious | not serious | serious | not serious | none | Moderate |

| **Outcome** | **Mirikizumab** | **Risankizumab** | **Relative effect (95% CrI)** | **Absolute effect (95% CrI)** | **Certainty** |
| --- | --- | --- | --- | --- | --- |
| PASI90 | 979/1379 (71.0%) | 788/1060 (74.3%) | OR 0.63 (0.01 to 39.37) | 97 fewer per 1,000 (from 715 fewer to 248 more) | Moderate |
| PASI75 | 1197/1379 (86.8%) | 413/462 (89.4%) | OR 0.59 (0.17 to 1.90) | 61 fewer per 1,000 (from 305 fewer to 47 more) | Moderate |
| PASI100 | 494/1379 (35.8%) | 468/1060 (44.2%) | OR 0.50 (0.03 to 7.06) | 158 fewer per 1,000 (from 418 fewer to 407 more) | Moderate |
| IGA/PGA/sPGA | 1049/1379 (76.1%) | 904/1060 (85.3%) | OR 0.59 (0.26 to 1.38) | 79 fewer per 1,000 (from 252 fewer to 36 more) | Moderate |
| AE | 749/1379 (54.3%) | 502/1060 (47.4%) | OR 1.00 (0.65 to 1.54) | 0 fewer per 1,000 (from 105 fewer to 107 more) | Moderate |
| SAE | 21/1379 (1.5%) | 23/1060 (2.2%) | OR 2.52 (0.36 to 20.08) | 31 more per 1,000 (from 14 fewer to 286 more) | Moderate |

## Profile 10. Mirikizumab compared to Tildrakizumab for plaque psoriasis

| **Outcome** | **No. of studies** | **Study design** | **Risk of bias** | **Inconsistency** | **Indirectness** | **Imprecision** | **Other considerations** | **Certainty** |
| --- | --- | --- | --- | --- | --- | --- | --- | --- |
| PASI90 | 0 | randomised trials | not serious | not serious | serious | not serious | none | Moderate |
| PASI75 | 0 | randomised trials | not serious | not serious | serious | not serious | none | Moderate |
| PASI100 | 0 | randomised trials | not serious | not serious | serious | not serious | none | Moderate |
| IGA/PGA/sPGA | 0 | randomised trials | not serious | not serious | serious | not serious | none | Moderate |
| AE | 0 | randomised trials | not serious | not serious | serious | not serious | none | Moderate |
| SAE | 0 | randomised trials | not serious | not serious | serious | not serious | none | Moderate |

| **Outcome** | **Mirikizumab** | **Tildrakizumab** | **Relative effect (95% CrI)** | **Absolute effect (95% CrI)** | **Certainty** |
| --- | --- | --- | --- | --- | --- |
| PASI90 | 979/1379 (71.0%) | 263/815 (32.3%) | OR 14.48 (0.34 to 877.51) | 551 more per 1,000 (from 183 fewer to 675 more) | Moderate |
| PASI75 | 1197/1379 (86.8%) | 517/815 (63.4%) | OR 3.02 (1.16 to 7.69) | 205 more per 1,000 (from 34 more to 296 more) | Moderate |
| PASI100 | 494/1379 (35.8%) | 89/726 (12.3%) | OR 1.45 (0.04 to 21.36) | 46 more per 1,000 (from 117 fewer to 626 more) | Moderate |
| IGA/PGA/sPGA | 1049/1379 (76.1%) | 469/815 (57.5%) | OR 2.45 (1.11 to 5.47) | 193 more per 1,000 (from 25 more to 306 more) | Moderate |
| AE | 749/1379 (54.3%) | 411/815 (50.4%) | OR 1.12 (0.73 to 1.69) | 28 more per 1,000 (from 78 fewer to 128 more) | Moderate |
| SAE | 21/1379 (1.5%) | 11/815 (1.3%) | OR 1.78 (0.23 to 16.56) | 10 more per 1,000 (from 10 fewer to 171 more) | Moderate |
