# Supplementary material for: Comparative efficacy and safety of IL-23 p19 monoclonal antibodies for plaque psoriasis: a bayesian network meta-analysis of randomized controlled trials
Source: Front Immunol. 2026 Aug 12;17:1873488. doi: 10.3389/fimmu.2026.1873488 (PMC13506889; doi:10.3389/fimmu.2026.1873488)
Supplement: Supplementary file 3 [file Table3.docx]

| **Section and Topic** | **Item #** | **Checklist item** | **Location where item is reported** |
| --- | --- | --- | --- |
| **TITLE** | | |  |
| Title | 1 | Identify the report as a systematic review. | Title page, title (identifies a Bayesian network meta-analysis of randomized controlled trials; consider adding ‘systematic review’ if required by the journal). |
| **ABSTRACT** | | |  |
| Abstract | 2 | See the PRISMA 2020 for Abstracts checklist. | Abstract, Objective, Methods, Results, and Conclusion. |
| **INTRODUCTION** | | |  |
| Rationale | 3 | Describe the rationale for the review in the context of existing knowledge. | Introduction, paragraphs 1–3. |
| Objectives | 4 | Provide an explicit statement of the objective(s) or question(s) the review addresses. | Introduction, final paragraph; Abstract, Objective. |
| **METHODS** | | |  |
| Eligibility criteria | 5 | Specify the inclusion and exclusion criteria for the review and how studies were grouped for the syntheses. | Methods, Sections 2.1 Study design, 2.3 Inclusion criteria, and 2.4 Exclusion criteria; study grouping described in Sections 2.5 and 2.8. |
| Information sources | 6 | Specify all databases, registers, websites, organisations, reference lists and other sources searched or consulted to identify studies. Specify the date when each source was last searched or consulted. | Methods, Section 2.2 Literature search strategy; databases searched: PubMed, Embase, Web of Science, and Cochrane Library; last search date: February 21, 2026; reference lists manually screened. |
| Search strategy | 7 | Present the full search strategies for all databases, registers and websites, including any filters and limits used. | Methods, Section 2.2 Literature search strategy; full database search strategies are stated to be provided in the Supplementary File. |
| Selection process | 8 | Specify the methods used to decide whether a study met the inclusion criteria of the review, including how many reviewers screened each record and each report retrieved, whether they worked independently, and if applicable, details of automation tools used in the process. | Methods, Section 2.5 Study selection and data extraction; two reviewers independently screened records and full texts; disagreements resolved by discussion or a third reviewer. |
| Data collection process | 9 | Specify the methods used to collect data from reports, including how many reviewers collected data from each report, whether they worked independently, any processes for obtaining or confirming data from study investigators, and if applicable, details of automation tools used in the process. | Methods, Section 2.5 Study selection and data extraction; two reviewers independently extracted data; disagreements resolved by discussion or a third reviewer. |
| Data items | 10a | List and define all outcomes for which data were sought. Specify whether all results that were compatible with each outcome domain in each study were sought (e.g. for all measures, time points, analyses), and if not, the methods used to decide which results to collect. | Methods, Section 2.6 Outcome definitions; outcomes sought: PASI75, PASI90, PASI100, IGA/PGA/sPGA 0/1, AE, and SAE. |
|  | 10b | List and define all other variables for which data were sought (e.g. participant and intervention characteristics, funding sources). Describe any assumptions made about any missing or unclear information. | Methods, Section 2.5 Study selection and data extraction; variables included author, year, study name, region, design, sample size, intervention, comparator, dose, follow-up, age, sex, efficacy outcomes, safety outcomes, and adverse events; assumptions for target dose arms and independent RCT units are described. |
| Study risk of bias assessment | 11 | Specify the methods used to assess risk of bias in the included studies, including details of the tool(s) used, how many reviewers assessed each study and whether they worked independently, and if applicable, details of automation tools used in the process. | Methods, Section 2.7 Risk of bias assessment; Cochrane RoB 2.0; two independent reviewers; disagreements resolved by discussion or a third reviewer. |
| Effect measures | 12 | Specify for each outcome the effect measure(s) (e.g. risk ratio, mean difference) used in the synthesis or presentation of results. | Methods, Section 2.8 Statistical analysis; dichotomous outcomes summarized as odds ratios with 95% credible intervals. |
| Synthesis methods | 13a | Describe the processes used to decide which studies were eligible for each synthesis (e.g. tabulating the study intervention characteristics and comparing against the planned groups for each synthesis (item #5)). | Methods, Sections 2.3–2.5 and 2.8; studies grouped by intervention, comparator, outcome, and target dose arm for each network synthesis. |
|  | 13b | Describe any methods required to prepare the data for presentation or synthesis, such as handling of missing summary statistics, or data conversions. | Methods, Sections 2.5 and 2.8; target dose arms extracted for multi-dose trials; independent RCTs within a single article analyzed as separate study units; event counts and sample sizes used for dichotomous outcomes. |
|  | 13c | Describe any methods used to tabulate or visually display results of individual studies and syntheses. | Methods, Section 2.8 Statistical analysis; network plots, forest plots, SUCRA curves, rankograms, heatmaps, and funnel plots described; Results, Figures 3–10. |
|  | 13d | Describe any methods used to synthesize results and provide a rationale for the choice(s). If meta-analysis was performed, describe the model(s), method(s) to identify the presence and extent of statistical heterogeneity, and software package(s) used. | Methods, Section 2.8 Statistical analysis; Bayesian network meta-analysis using MCMC; model choice based on convergence and heterogeneity. Software/package not reported in the manuscript. |
|  | 13e | Describe any methods used to explore possible causes of heterogeneity among study results (e.g. subgroup analysis, meta-regression). | Methods, Section 2.10 Heterogeneity analysis; Results, Sections 3.5–3.6; Figures 4–5. |
|  | 13f | Describe any sensitivity analyses conducted to assess robustness of the synthesized results. | Methods, Section 2.12 Sensitivity analysis; leave-one-out sensitivity analysis. |
| Reporting bias assessment | 14 | Describe any methods used to assess risk of bias due to missing results in a synthesis (arising from reporting biases). | Methods, Section 2.13 Publication bias assessment; contour-enhanced funnel plots and Egger’s regression test. |
| Certainty assessment | 15 | Describe any methods used to assess certainty (or confidence) in the body of evidence for an outcome. | Not reported. No formal certainty/confidence assessment (e.g., GRADE or CINeMA) is described in the manuscript. |
| **RESULTS** | | |  |
| Study selection | 16a | Describe the results of the search and selection process, from the number of records identified in the search to the number of studies included in the review, ideally using a flow diagram. | Results, Section 3.1 Literature search results; Figure 1. |
|  | 16b | Cite studies that might appear to meet the inclusion criteria, but which were excluded, and explain why they were excluded. | Results, Section 3.1 Literature search results gives categories and numbers of exclusions at full-text review; a citation list/table of excluded full-text studies is not reported in the manuscript. |
| Study characteristics | 17 | Cite each included study and present its characteristics. | Results, Section 3.2 Basic characteristics of included studies; Table 1 cites and summarizes each included study. |
| Risk of bias in studies | 18 | Present assessments of risk of bias for each included study. | Results, Section 3.3 Risk-of-bias assessment; Figure 2. |
| Results of individual studies | 19 | For all outcomes, present, for each study: (a) summary statistics for each group (where appropriate) and (b) an effect estimate and its precision (e.g. confidence/credible interval), ideally using structured tables or plots. | Results, Table 1 for study-level characteristics and group data; Results, Sections 3.7–3.8 and Figures 6–7 for effect estimates and precision. |
| Results of syntheses | 20a | For each synthesis, briefly summarise the characteristics and risk of bias among contributing studies. | Results, Sections 3.2–3.4 summarize characteristics, risk of bias, and evidence networks contributing to syntheses. |
|  | 20b | Present results of all statistical syntheses conducted. If meta-analysis was done, present for each the summary estimate and its precision (e.g. confidence/credible interval) and measures of statistical heterogeneity. If comparing groups, describe the direction of the effect. | Results, Sections 3.7–3.9 present all statistical synthesis results with ORs, 95% CrIs, and ranking estimates; Figures 6–8. |
|  | 20c | Present results of all investigations of possible causes of heterogeneity among study results. | Results, Sections 3.5–3.6 present transitivity and heterogeneity assessments; Figures 4–5. |
|  | 20d | Present results of all sensitivity analyses conducted to assess the robustness of the synthesized results. | Results, Section 3.10 Sensitivity analysis; Figure 9. |
| Reporting biases | 21 | Present assessments of risk of bias due to missing results (arising from reporting biases) for each synthesis assessed. | Results, Section 3.11 Publication bias assessment; Figure 10. |
| Certainty of evidence | 22 | Present assessments of certainty (or confidence) in the body of evidence for each outcome assessed. | Not reported. No certainty/confidence assessment for the body of evidence is presented. |
| **DISCUSSION** | | |  |
| Discussion | 23a | Provide a general interpretation of the results in the context of other evidence. | Discussion, first several paragraphs; comparison with mechanisms, pivotal trials, safety evidence, and prior network meta-analyses. |
|  | 23b | Discuss any limitations of the evidence included in the review. | Limitations, Section 5, especially limitations related to evidence quantity, imprecision, indirect comparisons, and incomplete outcome reporting. |
|  | 23c | Discuss any limitations of the review processes used. | Limitations, Section 5, including limitations related to network structure, RCT-only evidence, and lack of real-world data/cost-effectiveness. |
|  | 23d | Discuss implications of the results for practice, policy, and future research. | Discussion, Clinical implications; Conclusion, Section 6. |
| **OTHER INFORMATION** | | |  |
| Registration and protocol | 24a | Provide registration information for the review, including register name and registration number, or state that the review was not registered. | Methods, Section 2.2 Literature search strategy; PROSPERO registration number CRD420261382915. |
|  | 24b | Indicate where the review protocol can be accessed, or state that a protocol was not prepared. | Not reported. The manuscript provides the PROSPERO registration number, but does not state where the full protocol can be accessed. |
|  | 24c | Describe and explain any amendments to information provided at registration or in the protocol. | Not reported. Protocol amendments are not described. |
| Support | 25 | Describe sources of financial or non-financial support for the review, and the role of the funders or sponsors in the review. | Not reported. Funding/support statement should be added if required by the journal. |
| Competing interests | 26 | Declare any competing interests of review authors. | Not reported. Competing interests statement should be added if required by the journal. |
| Availability of data, code and other materials | 27 | Report which of the following are publicly available and where they can be found: template data collection forms; data extracted from included studies; data used for all analyses; analytic code; any other materials used in the review. | Not reported. Data availability, analytic code, and supplementary materials availability should be specified if required by the journal. |

*From:*  Page MJ, McKenzie JE, Bossuyt PM, Boutron I, Hoffmann TC, Mulrow CD, et al. The PRISMA 2020 statement: an updated guideline for reporting systematic reviews. BMJ 2021;372:n71. doi: 10.1136/bmj.n71. This work is licensed under CC BY 4.0. To view a copy of this license, visit <https://creativecommons.org/licenses/by/4.0/>
